# Passive acoustic monitoring (PAM) to assess benthic communities associated with offshore wind farms: insights and future directions

**DOI:** 10.1007/s10661-026-15614-5

**Published:** 2026-06-29

**Authors:** Laurence H. De Clippele, Ross J. Barnett, Marta Bolgan, Flora Kent, Kirsty Wright, Kate Brookes, Alexander Gilliland

**Affiliations:** 1 School of Biodiversity, One Health, and Veterinary Medicine, University of Glasgow, Graham Kerr Building, Science Way, Glasgow, G12 8QQ UK; 2 School of Biological and Environmental Sciences, University of Stirling, Stirling, UK; 3 Fugro, 1-9 The Curve, 32 Research Avenue North, Heriot-Watt Research Park, Edinburgh, UK; 4 Science, Evidence, Data and Digital, Marine Laboratory, Scottish Government, Marine Directorate, Aberdeen, UK; 5 Scottish Marine Energy Research (ScotMER) Programme Offshore Wind Directorate, Scottish Government, Aberdeen, UK

**Keywords:** Bioacoustics, Acoustic indices, Phonic richness, Fish, Invertebrates, Biodiversity

## Abstract

Understanding biodiversity in offshore benthic ecosystems is crucial as anthropogenic pressures like offshore wind development increasingly alter marine environments. Passive acoustic monitoring (PAM), widely used for marine mammal research, offers a promising yet underexplored tool for assessing broader faunal communities. Here, we investigate whether PAM data, collected initially for marine mammal monitoring, can reveal spatial variation in benthic biodiversity along Scotland’s east coast. We analysed passive acoustic data from eight offshore sedimentary habitats, identifying 16 distinct biological sound types likely produced by fish and invertebrates. Acoustic indices were also calculated and compared with environmental variables and infaunal benthic richness derived from open-source biodiversity databases. Our results show that key habitat variables, including substrate type, current velocity, and spawning suitability, drive variation in acoustic communities. The findings of this pilot study demonstrate that PAM can be used to detect biologically meaningful patterns in benthic assemblages, and with future work focusing on investigating longer-term datasets, it could offer a cost-effective tool for biodiversity monitoring across space and time. While several acoustic indices correlated with phonic richness and benthic diversity, we currently do not recommend their use in biodiversity monitoring. This study highlights the ecological value of existing acoustic datasets and advances our understanding of soundscape ecology and species-habitat relationships in changing marine environments.

**Supplementary Information:**

The online version contains supplementary material available at 10.1007/s10661-026-15614-5.

## Introduction

Marine biodiversity, the variety of life in the ocean, encompassing genes, species, communities, and ecosystems, is rapidly changing. This is due to both direct and indirect impacts of climate change and ocean sprawl, the proliferation of human-made structures in marine and coastal areas (Duarte, 2014). Monitoring marine biodiversity is critical to understanding these changes, sustaining ecosystem services, and guiding effective management of marine resources. To standardise biodiversity monitoring, the Group on Earth Observations Biodiversity Observation Network (GEO BON) proposed a set of essential biodiversity variables (EBVs), grouped into six classes: genetic composition, species populations, species traits, community composition, ecosystem structure, and ecosystem function (Pereira et al., 2013). Measuring the status and trends of these EBVs provides actionable information to support policy and management in the face of increasing human pressures and climate change (Muller-Karger et al., 2018).

The Intergovernmental Science-Policy Platform on Biodiversity and Ecosystem Services (IPBES) declared a global biodiversity emergency (IPBES, 2019), and it is therefore essential that effective long-term monitoring programmes are established to ensure sustainable development (Borja & Elliott, 2021). This is particularly important in an offshore marine environment, which can be difficult and expensive to access and where impacts are not always clearly visible (Nathanael et al., 2024). Due to the dynamic nature of marine ecosystems, it can be challenging to find clear relationships between human activities (and the associated pressures) and an ecosystem response. On the other hand, some interventions, if done correctly, can potentially enhance biodiversity and improve ecosystem outcomes (Strain et al., 2018). Therefore, monitoring methods should be able to detect when biodiversity increases as well as potential decreases as a route to demonstrating environmental gains, which is especially important in light of recent discussions around marine net gain (i.e. an approach to development in the marine environment that aims to leave the natural environment in a measurably better state than it was beforehand) (Hooper et al., 2021; Watson et al., 2025).

Traditional methods for monitoring the biodiversity living in, on, or near the seafloor (i.e. the benthos), include grab sampling, box cores, benthic trawls, and visual surveys (e.g. remotely operated vehicles, autonomous underwater vehicles, and time-lapse images/videos) (Scottish Government report, 2024). Except for time-lapse cameras attached to benthic observation stations and landers (Clark et al., 2024; De Clippele et al., 2023; Kutti et al., 2014; Osterloff et al., 2016), these approaches only provide a snapshot of information at one point in time. Some of these tools can introduce sampling biases and cause damage to sensitive features, such as slow-growing, long-lived, and/or structure-forming species and habitats (Beisiegel et al., 2017). There is a need, therefore, to improve monitoring regimes and investigate the effectiveness of non-invasive cost-effective tools, such as passive acoustic monitoring (PAM).

PAM is emerging as a tool to assess and monitor changes in aquatic benthic biological communities (Havlik et al., 2022; Minello et al., 2021). Traditionally, in marine ecosystems, PAM has been used to monitor the presence and abundance of marine mammals. However, in recent years, PAM has been proposed and used for the monitoring of fish and mobile invertebrates residing in benthic habitats at a high temporal resolution and for long periods (months to years) with global efforts rapidly increasing (Bolgan, 2025; Darras et al., 2025; Di Iorio et al., 2021; Havlik et al., 2022; Looby et al., 2023; Mooney et al., 2020; Van Hoeck et al., 2021). Sounds produced by marine fauna (e.g. marine mammals, and invertebrates such as fish, crustaceans, worms, and bivalves) contribute to the biophonical component of the acoustic landscape or soundscape, while, e.g. currents and wind contribute to the geophonical component, and boat noise and other man-made noise contribute to the anthrophonical component (Duarte et al., 2021; Goto et al., 2019). Characterising the biological component of underwater soundscapes and monitoring changes in acoustic communities’ composition, diversity, and abundance can provide information on animal presence and behaviour (i.e. mating, aggression), as well as on species diversity (Ladich, 1997; Fudge & Rose, 2009; Desiderà et al., 2019a; Mooney et al., 2020; Di Iorio et al., 2021). The acoustic community can be defined as the aggregation of soniferous species, and the phonic richness is a measure of the total number of types of sounds made by soniferous species (Lamont et al., 2022).

Both fish and crustaceans can produce either active or passive sounds (Caiger et al., 2020; Raick et al., 2021; Rountree et al., 2018). Active sounds are here defined as sounds which animals intentionally produce for communication purposes, i.e. all those sounds emitted by a sender which influence the behaviour of a receiver in a fashion that provides fitness-related advantages at least to the sender (Bolgan, 2025). For example, cod can vibrate their sonic muscles around their swim bladder, which produces “grunt” sounds used to attract mates during spawning events (Brawn, 1961) and spiny lobsters can make “rasp” stridulation sounds to fend off predators (Buscaino et al., 2011). Other sounds, which are here referred to as passive sounds, are non-intentional and can be a by-product of certain behaviours, such as air movement, jaw movement, swimming or food consumption (Looby et al., 2023; Parsons et al., 2022; Rountree et al., 2018). These sounds typically have more variable acoustic characteristics (Rountree et al., 2018). For example, scallops (*Pecten maximus*) produce a “cough” sound while moving water through their valves (Di Iorio et al., 2012). Some of these sounds may be more abundant during certain times of the day and year. The circadian cycles (i.e. the daily cycle) of acoustic activity are regulated by changes in light levels generated by the sun or the moon cycles (Feng & Bass, 2016).

Visually and aurally analysing the presence and abundance of different types of sounds can provide insight into patterns of marine biodiversity that can otherwise be difficult to monitor. A variety of acoustic metrics (i.e. acoustic indices, phonic richness, sound pressure levels) have been used as proxies for biodiversity and habitat condition (Mooney et al., 2020). Variations in the spatial or temporal patterns of these metrics can indicate changes in community composition, ecological state, and habitat condition (e.g. Desiderà et al., 2019 Sueur et al., 2019; Lamont et al., 2022). For example, variations in acoustic community composition have been linked to water depth and the percentage cover of structure-forming benthic habitats, such as coralligenous reefs, in the north-western Mediterranean (Di Iorio et al., 2021). Habitats regarded as healthy or in better condition generally exhibit higher phonic richness, although significant differences are not always detected (Lamont et al., 2022; Di Iorio et al., 2021).

To calculate the phonic richness, one needs to annotate all sound types. Because manually annotating all sound types in recordings is time-consuming, computational acoustic indices, originally developed for terrestrial habitats (Sueur, 2018), are increasingly being applied in marine environments (Pieretti & Danovaro, 2020; Minello et al., 2021; Lamont et al., 2022; Williams et al., 2022). Rather than investigating patterns of calls at the species or at the community level, acoustic indices (or soundscape indices) are used to calculate spatio-temporal changes of acoustic features (e.g. intensity, entropy etc.). They have also been proposed as useful tools for estimating changes in ecosystem function, since animals can rely on ambient sounds as sources of environmental cues for foraging, navigation, predation, or habitat selection (Sueur, 2018; Pieretti & Danovaro, 2020). Although multiple reviews have highlighted the potential of acoustic indices for biodiversity monitoring, they also note that further research is needed, as the relationship between traditional biodiversity measures, including phonic richness, and values derived from acoustic indices is not always straightforward (Minello et al., 2021; Mooney et al., 2020; Pieretti & Danovaro, 2020). In addition, noise pollution, equipment self-noise, fish chorusing, and even geophysical sounds can disrupt the reliability and interpretability of the indices (Parks et al., 2014; Minello et al., 2021; Nguyen Hong Duc et al., 2021). For example, equipment self-noise can artificially increase the acoustic complexity index (ACI) values, while fish chorusing can reduce it (Nguyen Hong Duc et al., 2021).

Given the projected expansion of offshore wind development around Scotland, there is a growing need to monitor the ecological impacts of these activities, including potential changes in marine biodiversity and ecosystem function. PAM and associated acoustic metrics offer a cost-effective, non-invasive approach to monitor these changes continuously over long periods, providing insights that are difficult to obtain from traditional survey methods. The Scottish Government has set a range of targets to cut greenhouse gas emissions, to be nature positive by 2030 (i.e. Scottish Biodiversity Strategy to 2045) and to generate more energy from renewable sources. The Climate Change (Scotland) Act 2019 commits the Scottish Government to reach net zero emissions of all greenhouse gases by 2045 (Reid, 2024). Offshore wind will play a vital part in meeting these targets and is set to expand substantially in Scotland over the next decade and beyond, as evident by the recent ScotWind seabed leasing round by the Crown Estate Scotland. In January 2021 and through its clearing process in August 2022, Crown Estate Scotland offered Option Agreements for 20 offshore wind projects around Scotland, totalling 27.6 Giga Watts (GW) of potential new offshore wind developments. The related offshore wind Innovation and Targeted Oil and Gas (INTOG) decarbonisation planning and leasing process outlined a further 5.4 GW of projects that have been offered exclusivity agreements.

Offshore developments typically occur in sedimentary habitats that are characterised by sandy, muddy, and coarse sediments. Previous acoustic studies usually focus on structurally complex habitats (i.e. such as coral reefs, seagrass meadows, rocky shores, oyster reefs), with sedimentary habitats mostly being used as a control rather than being the focus of a study (Ceraulo et al., 2018). Using PAM as a tool to monitor benthic habitats could be particularly relevant for the planned global offshore wind farm developments, as these have the potential to change benthic biodiversity at a local and regional scale through the introduction of hard substrates and changes in sediment deposition, food supply, and local hydrodynamics (Mangi, 2013; Clark et al., 2014; Dannheim et al., 2020). The application of PAM could provide long-term, remote, and scalable information on aquatic biodiversity, which would not be achievable by other monitoring techniques (Mooney et al., 2020; Palagyi et al., 2024; Ross et al., 2023; Van Parijs et al., 2021). However, there is a lack of evidence on the effectiveness of PAM as a technique for monitoring benthic communities, i.e. benthic and invertebrates in sedimentary habitats.

This study is a first step towards determining the value of PAM in benthic monitoring programmes and survey requirements for marine renewable energy devices (MREDs) in Scottish waters. Since changes in substrate composition are expected as a consequence of the installation of MREDs, the primary objective of this study was to determine if and how PAM can be used to detect spatial variation in benthic biodiversity in sedimentary habitats off the coast of Scotland. The acoustic community, phonic richness, and a range of acoustic indices were measured, and their usefulness for detecting spatial variation was assessed. The Marine Directorate has been using PAM to monitor cetacean species in Scottish waters over the past 10 years, with click detectors and broadband acoustic recorders deployed on moorings. As part of this study, we explore a small subset of this data to determine the potential to “unlock” data from this existing comprehensive PAM network to add value to Scottish marine monitoring programmes.

## Materials and methods

This study integrated multiple data sources to examine links between acoustic communities and benthic habitats. From selected moorings, passive acoustic monitoring (PAM) data formed the core dataset and were processed to derive acoustic community metrics, phonic richness, abundance, and acoustic indices. These acoustic data were paired with environmental variables (e.g. depth, temperature, currents, productivity) extracted from GIS layers (e.g. GEBCO, Bio-ORACLE, EUNIS) using spatial analysis tools in QGIS. Benthic biodiversity proxies were obtained from the OneBenthic model (faunal clusters and species richness), alongside habitat complexity metrics derived within a 10-km radius. Additional biological context was incorporated using fish spawning/nursery data (Marine Scotland, Franco et al., 2022) and species occurrence records from the NBN Atlas, which were cross-referenced with global databases of sound-producing species (GLUBS, WoRMS, FishSounds). All datasets were combined at the site level and analysed using multivariate statistical approaches (e.g. Bray-Curtis dissimilarity, ANOSIM, PERMANOVA, and RDA) to assess how environmental and biodiversity-related variables explain variation in acoustic communities.

### Study site selection

The study area lies within the exclusive economic zone of the UK, specifically along the east coast of Scotland. The depth range of the moorings was between 20 and 50 m. Due to the lack of permission to use data collected by windfarm companies, data from moorings located outside windfarms were selected to achieve this study’s objective. PAM metadata from 332 moorings provided by the Marine Directorate were screened for the presence of calibration information and broadband acoustic data (ECOMMAS, 2013). From the latter, 200 moorings were identified as having broadband and calibration data originating from 20 different locations. To understand if spatial and habitat-driven variation structures acoustic communities and to maximise the amount of data from contrasting habitat types while minimising the effect of seasons and interannual changes on the soundscape, the available data were searched for the year in which most sites had data collected in April. April was chosen as this is when key fish species are likely to spawn in Scottish waters (FishBase, 2024), and the effect of wind on the ambient noise would be relatively low (De Clippele & Risch, 2021).

**Fig. 1:**
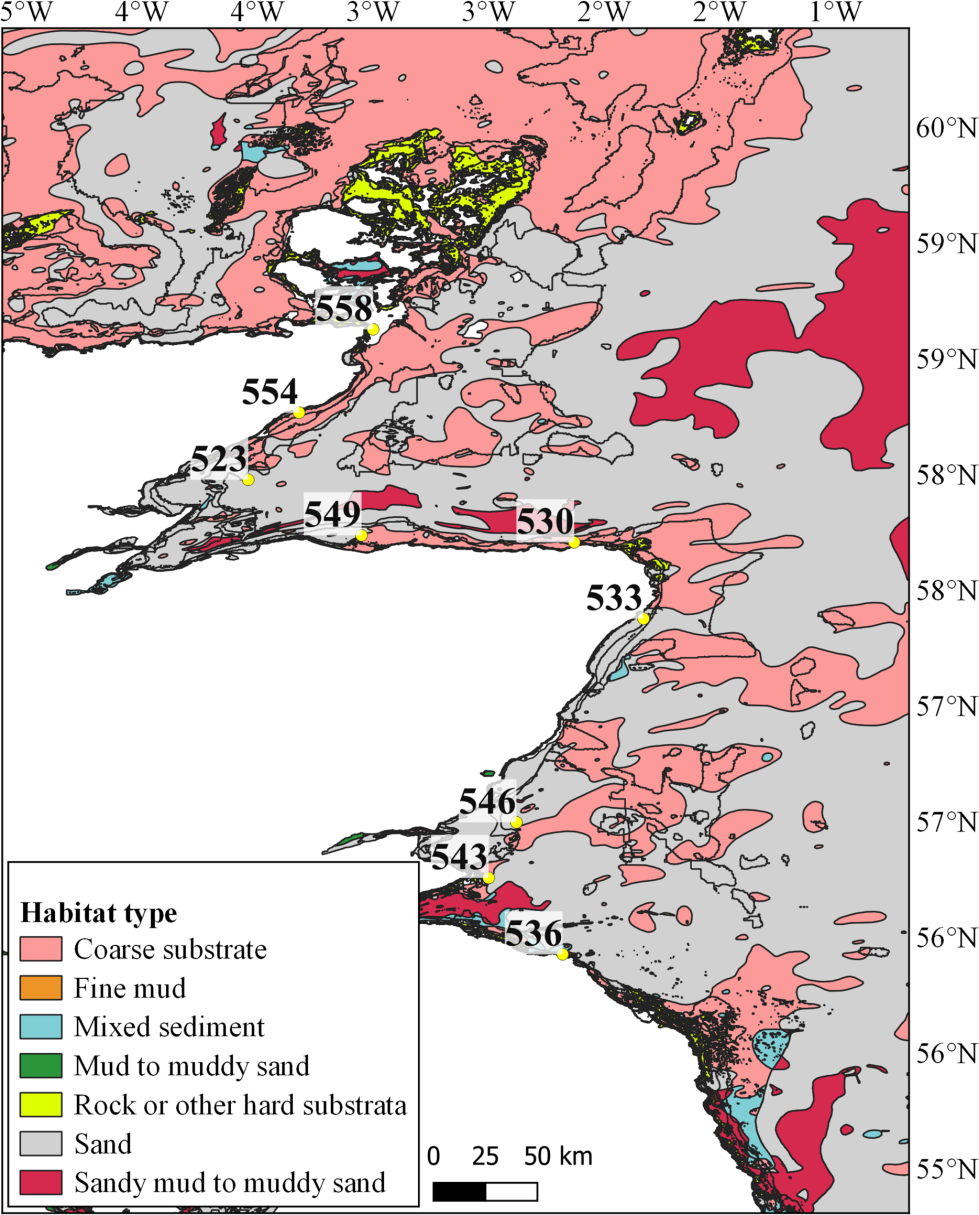
Map of the northeast (NE) of Scotland (UK) showing the locations of the passive acoustic mooring sites by deployment number and associated EUNIS habitat types. Site 554 is Latheron, 523 is Helmsdale, 549 is Spey Bay, 530 is Fraserburgh, 533 is Cruden Bay, 546 is Arbroath, 543 is St Andrews, and 536 is St Abbs. Site 558 is Duncansby Head, which was excluded from the analysis as it was heavily polluted with artificial mooring noise. Figure was created in ArcPro 3.4.0 © JNCC. Copyright and database right 2025. Contains data derived from the DEFRA Marine DEM (one arc second) © British Crown Copyright, 2025. All rights reserved. Permission Number: Defra012017.002. This product has been derived in part from material obtained from the UK Hydrographic Office with the permission of the UK Hydrographic Office, The Keeper of Public Records. NOT TO BE USED FOR NAVIGATION

The centre frequency of the 100 Hz 1/3 octave band sound pressure level (SPLrms) was calculated using PAMGuide (Merchant et al., 2015) to determine which day in April was the least affected by wind and anthropogenic noise pollution (De Clippele & Risch, 2021). The PAM data collected in April 2021 included most sites, 18 in total. The 100 Hz (1/3 octave band, SPLrms) data were plotted using ggplot in R version 4.0.0 and revealed that the 23rd of April was the most suitable day for further analysis, as it had the least amount of ambient noise at the 100 Hz 1/3 octave band (Supplementary Materials, SM1). However, one of the locations, the Arbroath site, only had data available until the 12th of April. To avoid reducing the number of sites for analysis, the 10th of April was identified as the second most suitable day. Visual inspection of historical weather data on visual crossing (visual crossing, 2024) revealed that the noise levels were predominantly driven by wind gust strength at the sites. Inspection of the acoustic data from the Latheron site confirmed that the peak in the 100 Hz 1/3 octave band SPLrms (up to ~ 130 dB re 1 µPa of the 1/3 Octave band) was related to ship activity very near the site (SM1). Of the 18 moorings sites reviewed, nine were used for further analysis as sharing permissions were in place during the duration of the project (Fig. 1). One of the locations, Duncansby Head (Deployment no 558), was heavily polluted with artificial mooring noise and was therefore also excluded, leaving eight sites in total (Table 1).

**Table 1:** Overview of the passive acoustic monitoring (PAM) datasets used in this study. The table provides the mooring’s ID number, deployment number, location name, and coordinates

ID	Deployment no.	Location and buoy number	Latitude (decimal degrees)	Longitude (decimal degrees)	Predicted habitat type
1	554	Latheron 5	58.27	−3.23	Coarse
11	549	Spey Bay 10	57.74	−3.05	Coarse
13	530	Fraserburgh 5	57.71	−2.13	Coarse
26	543	St Andrews 10	56.26	−2.5	Coarse
28	536	St Abbs 5	55.93	−2.18	Hard
6	523	Helmsdale 15	57.98	−3.54	Sandy
16	533	Cruden Bay 5	57.38	−1.83	Sandy
23	546	Arbroath 10	56.5	−2.38	Sandy

### Passive acoustic data

All recordings had a sampling rate of 96 kHz. All acoustic files were visually and auditorily screened in the software Raven Pro (version 1.6). The noise produced by vessels, the moorings, or pile driving was categorised as anthropogenic. These were assessed both aurally and visually, and cross-validated by acoustic experts at the Marine Directorate. Distinct anthropogenic noises such as pile driving and self or vessel noise were annotated in Raven with bounding boxes. These selections were then removed using the delete tool within Raven Pro, to further test if noise pollution affects acoustic index values (Zhang et al., 2024). Any biological sounds which may have been masked by this anthropogenic noise would also have been removed. Four subsamples of 10-min continuous recordings were selected per site, around sunset, midnight, sunrise, and midday. Different subsampling schemes have been adopted in previous studies (Desiderà et al., 2019; Harris et al., 2016); here, we relied on 10-min listening periods, which have been shown to correctly inform the variability present in the data (Desiderà et al., 2019). Based on historical data, sunrise happened at 06:16 and sunset happened at 20:08 on 10/04/2021; with the exception of St Abbs at dawn, these times appeared clear of shipping noise pollution. Due to slight differences in the timings of recordings at each site, the four selected 10-min recordings were the first after sunrise, the last before sunset, and the closest to both solar midday and midnight (SM3). The time difference between these timings varied between 5 and 30 min (SM3).

### Acoustic community, phonic richness, and abundance

Sounds likely produced by benthic fish and large invertebrates (< 2000 Hz) were manually annotated in Raven Pro version 1.6. The sound typology used to classify the types of sound was based on measurable acoustic features. Each dataset was annotated and subsequently re-checked in a second pass by the same annotator to ensure accuracy and internal consistency. For the purpose of this study, both active and passive sounds were considered. This resulted in the following: acoustic community (i.e. aggregation of different types of communicative and non-communicative sounds present in a recording), phonic richness (i.e. the total number of distinct sound types detected), and abundance (i.e. the total number of occurrences (counts) of specific sound types) data. The sounds’ envelope, as well as temporal and spectral variability across multiple replicates of a particular sound, can provide indirect information on whether a sound is passive or active. Communicative sounds can show variability in the spectral and temporal domain due to intrinsic (e.g. age (Pereira et al., 2020), motivation (Bolgan et al., 2020), fish size (Parmentier & Fine, 2016), hormonal level (Modesto & Canário, 2003), and extrinsic factors such as water temperature (Kever et al., 2015). A high variability in the sound envelope and in the temporal and spectral domain (i.e. high coefficient of variation) may indicate that the sound is passive rather than used for active communication (Rountree et al., 2018).

Due to the typically low-frequency content of fish sounds, spectrograms in Raven Pro were explored up to 4 kHz. However, some marine mammals can also produce sounds below 4 kHz, particularly baleen and some toothed whales (e.g. sperm whale, orcas), as well as seals. The sample rate was 96 kHz, the FFT size of 6400, and a frequency resolution of 15 Hz. Sounds with frequency components above 4 kHz were also annotated when they were visible in the lower frequency ranges (< 4 kHz), as some benthic invertebrate sounds can extend to around 10 kHz (e.g. Au & Banks, 1998; Goto et al., 2019). The *x*-axis was zoomed to ~ 12 s, to give enough detail to clearly label the start and end of specific acoustic events. Sounds were labelled and counted, and a datasheet was created containing information on the number of different types of sounds per subsample. Sounds could either be described as a pulsed or a frequency-modulated sound. A pulsed sound is a relatively brief, transient acoustic signal characterised by a structure made of discrete bursts of sound energy with sudden onset and offset (i.e. pulses), separated by silent intervals. A frequency-modulated sound is a continuous signal whose frequency changes over time, resulting in tonal variation (Erbe et al., 2022).

The following spectral metrics were measured: peak frequency (frequency with the highest energy), centre frequency (frequency that divides the spectral content into two intervals of equal energy), frequency 95% (frequency that divides the spectral content into two intervals containing 95% and 5% of the energy), and frequency contour percentile 25% (Freq 25%; frequency that divides the spectral content into two intervals containing 25% and 75% of the energy). Temporal metrics included sound duration (delta time; the interval from the onset of the first pulse to the offset of the last pulse), pulse period (time interval between the peaks of two consecutive pulses), and the number of pulses (number of sharp increases in acoustic intensity or cycles of amplitude modulation). All measurements were made in Raven for sounds with an adequate signal-to-noise ratio. Further definitions of these metrics are provided in Bolgan et al. (2020) and the Raven Pro 1.4 user manual.

### Acoustic indices

A total of seven acoustic indices (Table 2) were calculated (Sueur et al., 2014; Williams et al., 2022) for each of the 10-min subsamples. To calculate the acoustic indices, each selected recording was filtered using a short-term Fourier transform band-pass filter into different frequency bands at the low-frequency band (50–1000 Hz), a relatively higher frequency band (1000–2000 Hz), and this frequency band as a whole (20–2000 Hz). The chosen range of the different frequency bands was based on the frequency bounds of the annotated types of sounds. While peak frequency bands of some invertebrate sounds may not be included in this range, their sounds usually extend to the lower frequency ranges as well. As mentioned above, only sounds extending below 4 kHz were annotated. The indices were calculated in R using the packages *seewave* (Sueur et al., 2008) and *soundecology* (Villanueva-Rivera et al., 2018). Acoustic indices were calculated for both the raw and cleaned audio files, i.e. after mooring and anthropogenic noise were removed. The acoustic indices calculated from the cleaned audio files were standardised using the new recording length.

**Table 2:** Overview of the acoustic indices metrics used and indices calculated. Details on the R package used to calculate the acoustic indices are provided, including a brief description of how each is calculated (acoustic index descriptions are adapted from Sueur et al. (2014) and Williams et al. (2022))

Acoustic metrics	Abbreviation	R package	Description
Acoustic community	AC	Not applicable	The aggregation of different types of communicative and non-communicative sounds present in a recording, measured through labelling signals of interest in a spectrogram in Raven Pro
Phonic richness	PR	Not applicable	The total number of distinct sound types detected (Lamont et al., 2022). This was calculated in Excel, based on manual annotations conducted in Raven Pro
Abundance	A	Not applicable	The total number of occurrences (counts) of specific sound types, measured in Excel based on manual annotations conducted in Raven Pro
Acoustic complexity index	ACI	Seewave	Measures variability in intensity of frequencies across time. Designed to give high values where variable biophony is present, low values in systems with constant sounds
Acoustic evenness index	AEI	soundecology	Measures diversity across frequency bands. High values indicate greater unevenness between frequency bands. As diversity of sound increases, scores will decrease
Bioacoustic index	BI	soundecology	Measures cumulative intensity across frequency bands. Higher values indicate higher variability—intended to give proxies for avian species abundance
Total entropy	H	Seewave	Measures randomness across temporal and spectral domains. The calculation is adapted from Shannon’s diversity index. Higher values indicate more even signals (amplitude equally spaced across frequency and time). It is the product of TE and SE
Temporal entropy	TE	Seewave	Measures randomness across the temporal domain. Higher values indicate amplitude even across time bands within the recording
Spectral entropy	SE	Seewave	Measures randomness across the frequency domain. Higher values indicate amplitude even across frequency bands within the recording
Amplitude index	M	Seewave	Measures median of the amplitude envelope. This is a relative measure of sound amplitude. Higher values indicate louder recordings

### Environmental data

To understand what biological and environmental variables may contribute to explaining spatial differences in the soundscape, Geographic Information System (GIS) layers were downloaded from open-source platforms and imported into QGIS (version 3.4.13, QGIS, 2024). An overview of the data and their web links is provided in the supplementary materials (SM2) and includes depth extracted from the General Bathymetric Chart of the Oceans, the EUNIS (European Nature Information System) habitat type (Fig. 1) (British Crown Copyright, 2025). Temperature, current velocity, primary productivity, and dissolved organic carbon were extracted from the Bio-ORACLE platform. The QGIS spatial analyst tool (Extract Multi Values to Point tool) was used to extract the environmental information from the sites for statistical analysis.

### Biodiversity data from open-source platforms and literature search

All data were downloaded in spring 2024. Benthic biodiversity data were obtained from the OneBenthic portal (Cooper & Barry, 2017, 2020), which provides spatial predictions of 12 benthic faunal clusters together with associated cluster confidence layers (infaunal and epifaunal species). These clusters are derived from predictive random forest models trained on environmental variables and seabed core and sediment samples. Model development, validation procedures, and uncertainty assessments are described in detail in Cooper and Barry (2017, 2020). Each faunal cluster is associated with an assemblage of likely taxa that would be found in each area. For each mooring location, we extracted the following variables for statistical analysis: OneBenthic species richness, faunal cluster category, and the area (km^2^) of the cluster at that location. The in- and epifaunal OneBenthic species are invertebrates that are unlikely to make sound. However, they act as a proxy for habitat complexity and/or are food sources for sound-producing species that could produce either passive or active sounds.

To account for habitat diversity within the spatial scale relevant to local species, we calculated the number of faunal clusters present within a 10-km radius of each site, a measure hereafter referred to as “habitat complexity”. This radius was selected based on intermediate home ranges of species of interest: e.g. European Lobster typically has home ranges < 5 km (Smith et al., 2001), while cod may travel 15 km to find food (Neat et al., 2006). It is important to note that biological sounds may not travel over these distances with the sound’s frequency, amplitude, and the acoustic conditions of each habitat having an impact on detection distances. For example, sounds produced by a fish may travel further in warmer compared to colder water (Seri et al., 2023). The OneBenthic faunal cluster categories are also solely based on invertebrates, for which soniferous behaviour is largely unknown or unlikely (Looby et al., 2023; Parsons et al., 2022). We also extracted data on fish species which use our sites as essential spawning or nursery fish habitats from the essential fish habitat (Franco et al., 2022) and fisheries sensitivity maps (Marine Scotland, 2022). Areas in these maps are categorised as “absent”, “low”, and “high” for certain fish species. For the analysis, we counted the number of species categorised as “high” at each site.

To determine which soniferous species may be present locally, we obtained species occurrence records within a 10-km radius of each site (see above’s justification) from the National Biodiversity Network (NBN) Atlas (NBN Trust, 2024). The NBN Atlas contains species occurrence data, submitted by trusted organisations and recorders and collated, integrated and standardised by the NBN Trust (2024). The NBN data were filtered to include only relevant marine species, excluding historical, non-animal, and non-marine records from 2014 onwards to account for recent changes in species distribution, e.g. due to changing sea temperatures.

The resulting species list (De Clippele et al., 2025a) was compared against a recently published global inventory of species confirmed or expected to produce sound underwater as part of a collaboration between the Global Library of Underwater Biological Sounds (GLUBS) and the World Register of Marine Species (WoRMS) (Looby et al., 2023) and fishsounds.net. GLUBS assigns species to one of six categories reflecting knowledge of their sound-producing behaviour. A further literature search was then conducted to detail any available acoustic characteristics of these soniferous species, including, sound type, peak frequency, and duration.

### Statistical analysis

To determine what drives the differences between the acoustic communities, first, the acoustic community data were Hellinger transformed using the *decostand* function in the *vegan* package (Oksanen et al., 2017). A Hellinger transformation was used to convert sound type abundance values to relative values, to address absences of certain types of sounds across multiple samples. Then, the Bray-Curtis dissimilarity measure was computed with the *vegdist* function. This measure calculates the dissimilarity between two samples based on the relative abundances of species present in those samples (Oksanen et al., 2017). To test if the acoustic communities significantly differ in relation to the OneBenthic faunal cluster type and the habitat type groups, an analysis of similarity (ANOSIM) was conducted using the Bray-Curtis dissimilarity matrix. ANOSIM compares the mean of ranked dissimilarities between groups to the mean of ranked dissimilarities within groups. This was followed by a permutational analysis of variance (PERMANOVA) to test the magnitude of the dissimilarities, using the *adonis2* function. This allowed to test if the patterns of variation in composition and in abundance of types of sounds in relation to faunal cluster and habitat type were different. To test for collinearity among explanatory variables, Pearson’s correlation tests were conducted using the *cor.test* function in R (Best & Roberts, 1975; Hollander & Wolfe, 1973). Variables with strong correlations (> 0.8) were removed. If differences were found using ANOSIM, then similarity percentage (SIMPER) analysis, using vegan’s *simper* function, was used to identify which types of sounds primarily accounted for observed differences in soundscape assemblages between faunal clusters and habitat types. This method does this by calculating the average percentage contribution of each variable to the overall Bray-Curtis dissimilarity between the chosen groups.

This was followed by the development of multivariate redundancy analysis (RDA) models to establish which variables contributed to explaining differences in the acoustic communities. The explanatory variables (i.e. depth, temperature, current speed, primary productivity, dissolved organic carbon, substrate, OneBenthic richness, Area of the OneBenthic class, the number of species that use the area as a nursery or for spawning, and time of day) were included and/or normalised using the *decostand* function in the *vegan* package. This function makes the margin sum of squares equal to one (default margin is 1) (Oksanen et al., 2017). Note that OneBenthic richness is derived exclusively from invertebrate assemblages, which themselves are not known to produce audible sounds. Instead, this metric serves as a proxy for habitat complexity or functioning, which may, in turn, coincide with higher abundance and diversity of soniferous species. To determine which explanatory variables were statistically important, forward selection was conducted using the *ordistep* function. The selected variables were then used in a partial RDA model, constraining the variables that are not metrics representative of the benthic biodiversity. RDA was chosen because it allows partitioning of variance among sets of predictors, enabling assessment of the relative contribution of environmental versus biodiversity-related variables. As the purpose of this analysis was explanatory rather than predictive, model training/validation or data-splitting procedures were not applicable, and data exploration was limited to normalisation, correlation checks, and forward selection of predictors. Correlation and Kruskal-Wallis tests were furthermore used to test correlations between metrics and differences between habitat types and faunal clusters. To test correlations between the habitat types and the acoustic indices, the habitat types were categorised, with “2” indicating coarse/hard substrates and “1” indicating sandy substrates.

## Results

### Benthic biodiversity

**Fig. 2:**
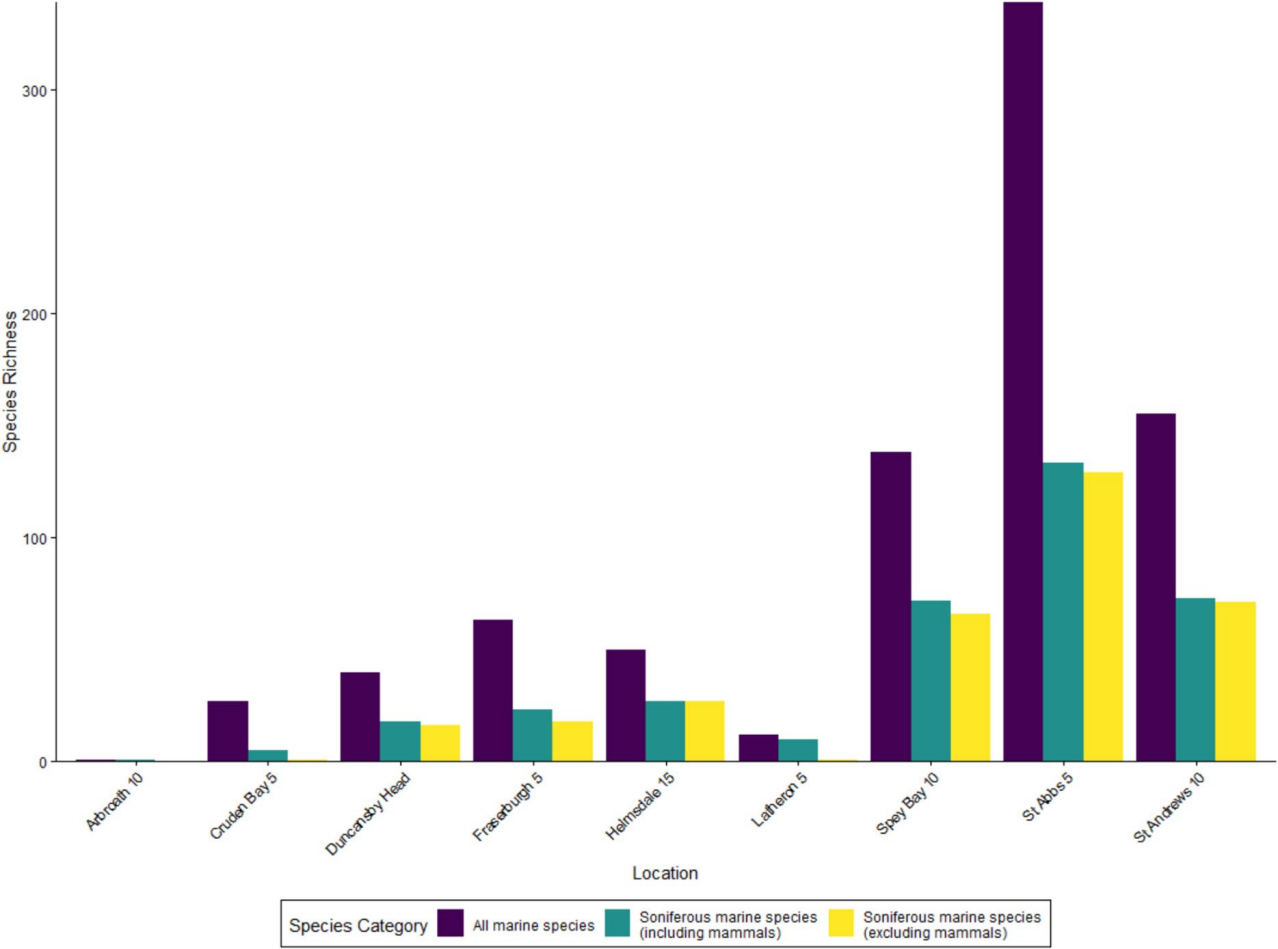
Bar chart denoting the number of species from NBN data found in the study locations in each of the GLUBS categories. Bars are coloured according to the Class of each animal species. Figure was created in Rv4.5.1

The data extracted from the NBN database resulted in a total of 5615 records belonging to 482 different species, extracted from the 10-km^2^ buffer area around the location of the mooring sites. The species list from NBN Atlas across all eight sites was combined with the publicly available soniferous information from the GLUBS database. Of the 482 species recorded in the NBN data, 49 species had been assessed in the GLUBS database (Looby et al., 2023; Parsons et al., 2022). Details of the soniferous categories and numbers of species in different taxonomic groups are presented in Table 3 and Fig. 2 and can be downloaded from Zenodo (De Clippele et al., 2025a). Of the 433 species not featured in the database, 417 were invertebrates, which are understudied in terms of the sounds they produce, so should be treated as “unknown or undetermined”. Other species that were not assessed were two *Elasmobranchii* (shark) species and 14 *Actinopterygii* (ray-finned fish) species. The full list of species found in the buffer area includes invertebrate and vertebrate species, as well as all species included in Marine Scotland’s Essential Fish Habitat report (Franco et al., 2022), some of which were not recorded in the NBN records (De Clippele et al., 2025a). Because the NBN records are not collected in a standardised manner, this data is not used in the analysis but is used as contextual information.

**Table 3:** Overview of the types of sounds (i.e. active, passive, unconfirmed, or unlikely) the 49 species make according to GLUBS database. The number of species in each category within higher taxonomic groups is provided, along with the names of highlighted species in each category also provided. More detail can be found in the “Soniferous species review” document, which can be downloaded from Zenodo (De Clippele et al., 2025a)

Group	Type of sound	Highlighted species
Mammals	**Active sound** = 10 species	Grey seal (*Halichoerus grypus*), harbour seal (*Phoca vitulina*) and eight cetaceans
Fish	**Do not or unlikely to make sound** = 7 species	Angler (*Lophius piscatorius*), dragonets (*Callionymus lyram*, *C. reticulatus*), common sole (*Solea solea*), lemon sole (*Microstomus kitt*), plaice (*Pleuronectes platessa*)
	**Likely to make sound but unconfirmed** = 16 species	Atlantic mackerel (*Scomber scombrus*), others
	**Passive sound** = 6 species	Conger eel (*Conger conger*), pollock (*Pollachius pollachius*), others
	**Active sound** = 10 species	John dory (*Zeus faber*), Atlantic cod (*Gadus morhua*), others
	**Active and passive sound** = 1 species	Corkwing wrasse (*Symphodus melops*)
Invertebrates	**Unknown to make sound** = 116 species	Brittlestars, spiny starfish (*Marthasterias glacialis*)
	**Passive sound** = 3 species	Urchins, scallops
	**Active sound** = 2 species	Lobsters
	**Undetermined to make sound** = 113	Prawns, shrimp, crabs, bivalves, jellyfish, sea cucumbers, anemone, hydroids, sponges, squid, starfish, worms

### Environmental data

A total of four PAM moorings were located within “coarse” sediment habitat types (i.e. Spey Bay, Latheron, Fraserburgh, St Andrews), one in “rock or hard” substrate (i.e. St Abbs) and three within “sandy” habitat types (i.e. Cruden Bay, Arbroath, Helmsdale). The coarse and hard substrate categories were merged as “hard substrates” for further analysis. The depth of the moorings ranged from 21 to 48 m depth, with Arbroath (47 m) and Helmsdale (48 m) being the deepest sites, and Spey Bay (23 m) and Cruden Bay (21 m) being the shallowest sites. The velocity ranged from 0.030 to 0.112 m s^−1^, with Helmsdale having the lowest and Fraserburgh the highest velocity. DOC ranged from 262.774 to 285.318 mmol m^−3^ and PP from 0 to 0.007 g m^−3^ day^−1^, with Fraserburgh having the lowest and Spey Bay the highest DOC and PP. The temperature ranged from 8.535 to 9.638 °C, with St Abbs having the lowest and Spey Bay the highest. More details on the environmental variables per site can be found in SM4 Table 4. Only three benthic faunal clusters were found to be associated with the study locations, i.e. D2a, D2b, and D2c. According to Cooper and Barry (2017), the D2a assemblages are found in areas with more mixed sediments, while assemblages associated with D2b and D2c are found associated with deeper water and are assumed to be largely stable through time. The D2b assemblage is found in areas of high mud content with weaker currents and D2c dominates in areas of higher sand content. Polychaetes were the dominant faunal group across the assemblages, with bivalve molluscs being a feature of group D2b. D2c has the lowest mean number of taxa and abundances associated with faunal groups (Table 4).

**Table 4:** Dominant benthic faunal groups per cluster, along with the dominant families according to the model produced by Cooper and Barry (2017)

Faunal cluster type	Sites	Taxa	Family
D2a	Latheron	Five polychaeta (P) and one nemertea (N) taxa	Spionidae (P), Glyceridae (P), Nemertea (N), Terebellidae (P), Capitellidae (P), Phyllodocidae (P)
D2b	Fraserburgh, St Andrews, Arbroath, Helmsdale	Seven polychaetes (P), one echinoderm (E), one nemertea (N) and one bivalve mollusc (BM) taxa	Spionidae (P), Amphiuridae (E), Nephtyidae (P), Lumbrineridae (P), Oweniidae (P), Cirratulidae (P), Capitellidae (P), Nemertea (N), Semelidae (BM), Ampharetidae (P)
D2c	Spey Bay, St Abbs, Cruden bay	Three polychaete (P) taxa	Nephtyidae (P), Spionidae (P), Opheliidae (P)

The faunal cluster’s habitat size underneath the moorings located at Helmsdale, St Andrews, and Cruden Bay was the largest. The “complexity”, i.e. the number of different classes found per mooring site, within a 10-km^2^ radius is shown in the Supplementary Materials (SM4). The most complex site was St Andrews, with 6 faunal cluster classes ranging from 1.25 to 154.45 km^2^ in patch size. The least complex sites were Latheron and Fraserburgh, each only having 3 benthic classes present, although both of these sites were in the bottom four for sea surface area within the 10 km^2^ buffer. This is because they were located close to land, meaning that some of the 10 km^2^ overlapped with land rather than the sea. All other sites had 5 classes present, though many had less than 0.5 km^2^ of some classes. It is noteworthy that all sites, except Helmsdale, were located within 10 km from land meaning that part of their 10-km^2^ buffer zone did not contain any benthic classes.

Some of the locations were categorised as potential spawning grounds for cod, plaice, sandeel and whiting, and nursery areas for cod, spurdog, blue whiting, tope shark, herring, hake, ling, mackerel, anglerfish, plaice, sandeel, and spotted ray (Franco et al., 2022). An overview of which species were listed to use each of our study locations as a spawning or nursery habitat is available on Zenodo (De Clippele et al., 2025a).

### Acoustic community

In total, 2592 individual sounds were labelled across the 310 min of audio examined. These sounds were labelled and categorised as 16 different types of sounds, likely including both passive and active sounds (Table 5). Detailed descriptions of each of the sounds’ acoustic characteristics can be found in the supplementary materials (SM5), which can be used for future identifications. Spey Bay and St Andrews had the highest abundance of sounds across the four 10-min samples. Two types of sounds were characterised as active sounds, as their acoustic features were similar to those previously reported for communicative fish sounds (Desiderà et al., 2019; Bolgan et al., 2022; Raick et al., 2023b); these were labelled “thump” and “drum”. Thump was recorded in Arbroath, Spey Bay, St Abbs, and St Andrews, while drum was only recorded in Spey Bay (Table 5). “Thump” can be described as a complex sound made of two components: a pulsed component followed by a frequency-modulated component. Its total abundance was highest at St Andrews. “Drum” can be described as a multipulse series and was present only at Spey Bay. The types of sounds “whoop”, “tap”, “sneeze”, “whew”, and “plop” were likely either fish or invertebrate active or passive sounds, while the others, such as “tick”, “grunt”, “scrape”, “crackle”, “click”, “squeak”, “snap”, and “chirp” were likely either invertebrate active or passive fish sounds (Table 5).

**Table 5:** Overview of the EUNIS habitat type, OneBenthic faunal cluster number, the total number of occurrences per sound type (ST), and the average ± standard deviation of the phonic richness per site. The acoustic characteristics and spectrograms are provided in SM5 and SM6

	Latheron	Fraserburgh	St Andrews	Spey Bay	St Abbs	Helmsdale	Arbroath	Cruden Bay
Habitat type	Coarse	Coarse	Coarse	Coarse	Rock or hard	Sandy	Sandy	Sandy
Faunal class	D2a	D2b	D2b	D2c	D2c	D2b	D2b	D2c
ST1—thump	3	0	66	50	19	0	5	0
ST2—tick	5	4	11	21	2	0	2	4
ST3—grunt	3	0	0	7	1	10	1	0
ST4—scrape	16	0	0	5	0	1	31	0
ST5—whoop	4	0	0	0	0	0	0	0
ST6—tap	15	5	224	499	108	3	23	47
ST7—crackle	9	0	0	0	25	0	0	0
ST8—squeak	3	0	0	0	0	0	0	0
ST9—sneeze	1	0	0	0	0	0	0	0
ST10—snap	1	0	0	2	0	0	0	0
ST11—chirp	1	0	4	11	1	0	1	0
ST12—whew	1	0	0	2	0	1	0	1
ST13—plop	1	0	3	9	0	0	5	0
ST14—whoosh	0	0	0	1	0	0	1	0
ST15—hard grunt	0	0	0	1	1	0	0	0
ST16—drum	0	0	0	15	0	0	0	0
Total number of sounds	**63**	**9**	**308**	**623**	**157**	**15**	**69**	**52**
Av. phonic richness per sample	**3.00 ± 0.76**	**1.00 ± 0.00**	**2.50 ± 0.53**	**5.25 ± 1.16**	**2.33 ± 0.52**	**1.00 ± 0.76**	**2.50 ± 1.60**	**1.25 ± 0.46**
Total phonic richness	**13**	**2**	**5**	**12**	**7**	**4**	**8**	**3**

The acoustic community data exhibited distinct clusters depending on the habitat type (i.e. hard vs sandy habitats) (ANOSIM: R 0.1426, *p*-value < 0.02) and faunal cluster type (i.e. D2a,b,c) (ANOSIM: *R* = 0.12, *p*-value = 0.05). There was a significant interaction of faunal cluster and habitat type (PERMANOVA; *F* = 3.1173, *p*-value = 0.002). The cumulative contribution of the most influential types of sounds between hard and sandy substrates was “thump” (73%), “tick” (63%), and “grunt” (52%) with “thump” and “grunt” being higher in hard substrates and grunt being higher in sandy habitats. “Thump” and “crackle” explain the majority of the dissimilarity between D2b and D2a (ST1 74.85% and ST7 67.76%) and “grunt” and “thump” between D2b and D2c (ST3 79.61% and ST1 68.00%). “Whoop” (74.34%) and “thump” (68.22%) are the other two types of sounds which were most different between D2b and D2a. “Thump” and “tick” were on average highest in D2c, “grunt” was on average highest in D2a, although only one site was associated with this faunal cluster. “Whoop” was only present in D2a (Table 5). Since we were not confident in attributing the recorded sound types to specific species, sounds were analysed and classified at the level of sound types rather than species identity (e.g. Desiderà et al., 2019; Di Iorio et al., 2021).

**Fig. 3:**
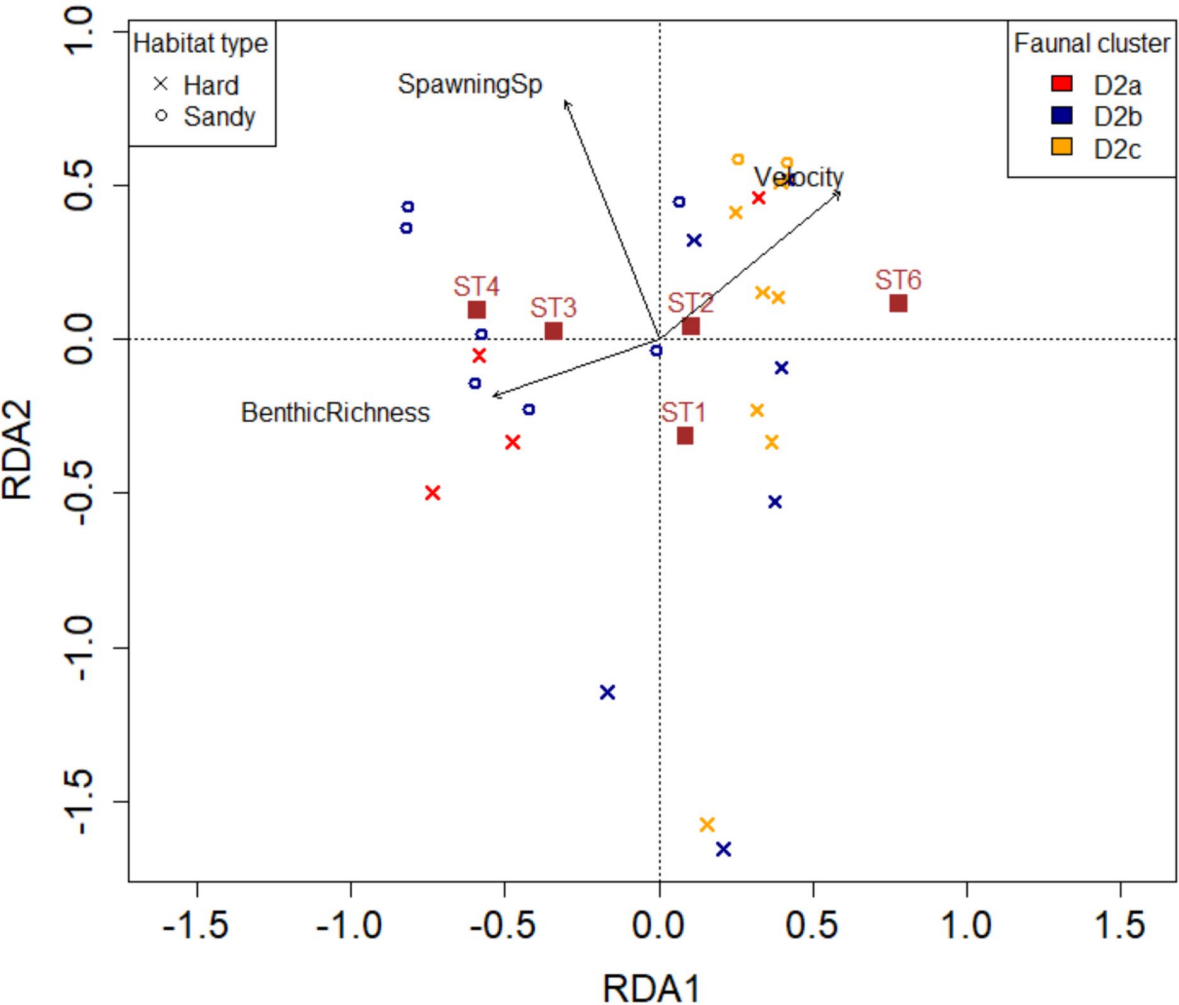
RDA plot showing the relationship between the acoustic communities and biodiversity and environmental variables, highlighting patterns of variation and key drivers of differentiation. ST1 is “Thump”, ST2 is “Tick”, ST3 is “Grunt”, ST4 is “Scrape”, ST6 is “Tap”. Figure was created in Rv4.5.1

An RDA was conducted to establish which environmental and biodiversity variables drive differences in the acoustic communities (i.e. based on abundance data of the different types of sounds) associated with each of the samples (Fig. 3). Benthic richness, velocity, and the number of spawning species were kept in the final model. The model explained 27% of the variation in the acoustic communities. A partial RDA, constraining velocity, showed that the benthic biodiversity variables (i.e. NBN benthic richness and number of spawning species) accounted for 22% of the variation, while velocity accounted for 5%. A total of 73% of the variability remains unexplained. ST6 “tap”, ST1 “thump”, ST1 “Tick”, ST4 “scrape”, and ST3 “grunt” were the top five sounds driving differences between the different communities.

Removing noise pollution from recordings resulted in files of different lengths (SM3). Latheron, Arbroath, and Fraserburgh had the highest occurrence of anthropogenic noise, based on the removal of distinct anthropogenic noise events. Helmsdale was the least noise-polluted site, with only one second being removed on average. The dawn recording from St Abbs was listened to; however, no sounds were labelled due to loud boat noise masking all other audio in this recording.

### Phonic richness

**Fig. 4:**
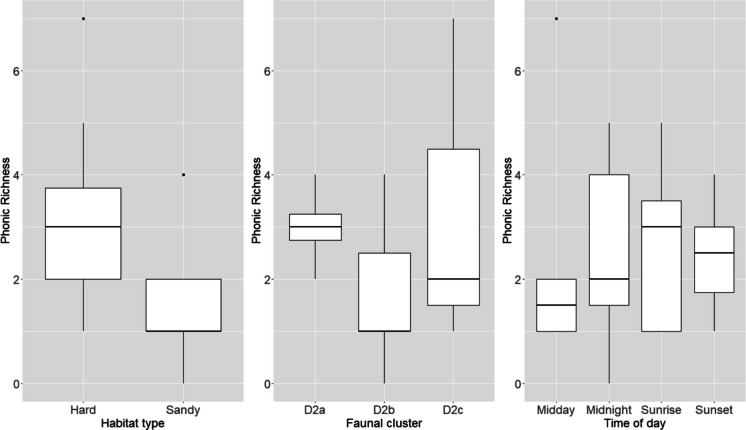
Boxplots showing how the phonic richness relates to the EUNIS habitat type, OneBenthic fauna, and the time of the day. Figure was created in Rv4.5.1

While Spey Bay, Latheron, and Arbroath had the highest total phonic richness, Spey Bay, Latheron, and St Andrews had the highest average richness of types of sounds (Table 5). Overall, the phonic richness (PR) was significantly higher in areas with coarse/hard EUNIS habitat types, compared to sandy EUNIS habitat types (Kruskal-Wallis test, *p*-value = 0.03; Fig. 4). When comparing the PR between the benthic faunal clusters, no significant differences were found (Fig. 4). PR at sunrise and sunset tended to be higher compared to midday and midnight, although no significant differences between time of the day could be found, likely due to the lack of temporal replicates per site (Fig. 4). A negative relation (*p*-value < 0.05) between phonic and benthic richness was found (correlation −0.30), although this was not significant.

### Acoustic indices

Three of the acoustic indices (definitions and acronyms are explained in Table 1) were correlated with benthic richness, i.e. ACI, AEI, and M (Table 6). Higher benthic richness values were correlated with a higher acoustic complexity index, a lower unevenness between frequency bands, and quieter recordings (Table 6).

**Table 6:** Overview of the relationships between the traditional metrics of benthic habitats and the computational acoustic metrics. Only significant (*p* < 0.05) Pearson correlation values are shown in this table

Traditional metrics	Acoustic index	Correlation values
OneBenthic richness	ACI low	+ 0.38
	ACI high	+ 0.36
	ACI broad	+ 0.38
	AEI broad	−0.39
	M high	−0.38
Habitat type (i.e. hard vs soft sediments)	BI low	−0.56
	H low	+ 0.53
	H broad	+ 0.50
	TE low	−0.38
	TE broad	−0.37
	SE low	+ 0.54
	SE broad	+ 0.51
	M high	+ 0.54
Phonic richness	BI low	−0.37
	H low	+ 0.53
	H broad	+ 0.51
	TE low	−0.38
	TE broad	−0.37
	SE low	+ 0.54
	SE broad	+ 0.51
	M high	+ 0.54

A total of five acoustic indices were correlated to habitat type and phonic richness, i.e. BI, H, TE, SE, and M. Harder substrates were correlated to more even signals as a whole, but not across time bands within the recordings. Harder substrates were also related to louder recordings, but a lower variability across frequency bands. Since phonic richness is correlated to harder substrates, the acoustic indices followed a similar pattern (Table 6).

**Fig. 5:**
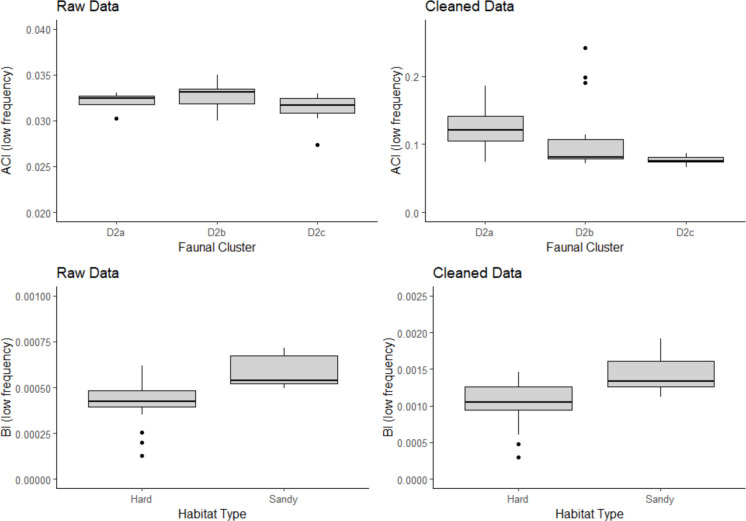
The acoustic complexity index (ACI) and bioacoustic index (BI) calculated for the low-frequency band against the faunal cluster classes and habitat type for the uncleaned raw and cleaned (i.e. which had noise pollution removed) recordings. Figure was created in Rv4.5.1

The abovementioned indices, calculated from the cleaned data, were compared with those calculated from the raw data to test if they produced different results. No overall significant differences were found when comparing the index values with each other; however, Fig. 5 shows examples of how ACI and BI differ depending on them being calculated on raw or cleaned data.

## Discussion

This pilot study demonstrates that spatial variation in habitat type and benthic richness in offshore environments can be detected using acoustic community composition and phonic richness derived from PAM data originally collected for marine mammal surveys. Significant differences in the acoustic community composition and the phonic richness were found in relation to the benthic faunal cluster classes and their habitat types, with benthic richness and the number of spawning species being important drivers in explaining differences between the communities. While significant differences for computationally efficient acoustic indices were also found, i.e. some acoustic indices indicated that harder substrates were associated with higher sound levels and that higher benthic richness was associated with greater acoustic complexity, we do not recommend that their use be implemented, which has also been stated by Sugai et al. (2026). To understand changes in species-specific sounds or the acoustic community as a whole and to understand which acoustic community metrics are relevant for management, data collected over longer time periods and across changing noise conditions are needed.

### Acoustic community

This study shows that in sedimentary habitats, acoustic communities are broadly associated with differences in benthic richness and habitat types. The acoustic community and richness of the two EUNIS habitat types studied (coarse/hard and sandy habitats) were different. Two diversity variables and one environmental variable explained the differences in the acoustic community composition best. Of the diversity variables, benthic richness and the area’s suitability for spawning for a larger number of fish were found to be the most important. This result aligns with previous research, which has demonstrated that acoustic community properties, such as phonic richness and acoustic abundance (i.e. the number of sounds per type), closely reflect fish community characteristics, including species richness and abundance, as measured by traditional methods like underwater visual census (Desiderà et al., 2019). The environmental driver that was important in explaining differences between the acoustic community was the current velocity. Local differences in current speed could indeed impact the soundscape indirectly through their known impacts on benthic biodiversity and substrate composition, including the range of sound detectability (Cooper & Barry, 2017, 2020; Kregting et al., 2016). If multiple or multi-channel hydrophones are used in combination with image or video data (Mouy et al., 2023; Dantzker et al., 2025), species-specific sounds can be identified, while potentially also estimating the source level and detection distances, which could help account for any confounding effects.

Nonetheless, a substantial proportion of the variation in acoustic community composition remained unexplained by the RDA models, suggesting that incorporating finer-scale biodiversity metrics and additional environmental variables could improve explanatory power and ecological interpretation. This study relied on using open-access coarse resolution GEBCO (~ 500 × 500 m) and EMODNet (~ 100 × 100 m) data. In the context of offshore developments, windfarm companies could contribute to improving ecological interpretation by collecting fine-scale environmental data through remotely operated vehicle surveys, grab and box-core sampling, high-resolution multibeam surveys, and the deployment of benthic landers. Benthic landers can be equipped with hydrophones, time-lapse cameras, and other sensors that collect information on current speed, temperature, and salinity changes (e.g. as in De Clippele et al., 2025a, 2025b).

Although analysing acoustic community data is time-consuming, it can allow for an in-depth understanding of which types of sounds are more or less common in different habitats or can be used as an indication of habitat condition. Understanding which acoustic communities are associated with certain habitats can be useful to monitor the impacts of degradation or restoration over time (Lamont et al., 2022). If a habitat were to change, e.g. from sandy to hard, then this would likely impact the benthic community composition. Likewise, if key predators, who are part of the benthic community, were to disappear due to, e.g. disease or over-harvesting, then this would also likely be detected in the soundscape if these predators are soniferous or prey on soniferous species. While in such a scenario, changes in the in and epifauna may happen at different timescales, early detection could inform management of upcoming longer-term changes. If landers were used and deployed for continuous monitoring, these long-term datasets could then be used to differentiate natural diel and seasonal patterns from climate change or human-driven changes and therefore act as early-warning tools to inform management (De Clippele et al., 2025b).

### Phonic richness

Here we demonstrate that phonic richness, encompassing both communicative and non-communicative sounds, can serve as a proxy for sedimentary habitat types and therefore habitat heterogeneity. While offshore development projects (e.g. windfarms and other MREDs; Dannheim et al., 2020) routinely incorporate detailed substrate mapping, acoustic metrics provide complementary information that extends beyond physical habitat classification. Specifically, acoustic monitoring captures the biological activity associated with habitat heterogeneity, reflecting not only structural complexity but also the presence, behaviour, and temporal dynamics of sound-producing taxa. As such, acoustic metrics could detect ecological richness and functional use of habitats in ways that mapping alone cannot, offering an additional, non-invasive tool for monitoring changes in benthic communities in response to offshore developments.

Overall, the phonic richness was higher for coarse/hard EUNIS modelled habitat types, which is in agreement with studies conducted in the Mediterranean (Ceraulo et al., 2018; Di Iorio et al., 2021). Unless reef-forming tube worms or bivalves are present within a soft-bottom habitat, coarse and hard sediments are generally found to be associated with higher biodiversity compared to sandy-soft bottoms (Coolen et al., 2015; Cooper & Barry, 2017, 2020; Lillis et al., 2014), which is also reflected in this study’s phonic richness values. Ceraulo et al. (2018) compared the acoustic communities associated with a seagrass meadow and sandy bottom habitats and found that the acoustic richness was higher in seagrass habitats. Hard/coarse habitats provide surface areas for sessile epifauna to attach to and have a higher availability of microhabitats, which in turn increase the niches available for species, with the data in Fig. 4 being in line with this assumption. While not enough spatial replicates were available to test if all faunal clusters were significantly different, the cluster (D2b) characterised by muddy sediments had the lowest phonic richness. The cluster represented by mixed sediments (D2a) had the highest phonic richness, while those with sandy habitats (D2c) were the most variable and had an intermediate average phonic richness (Fig. 4).

Sandy habitats are dynamic and mobile environments which allow animals to burrow to ambush prey or escape predators (Kenyonax et al., 1995; Steendam et al., 2020), which could affect their acoustic detectability. In addition, certain species, such as sandeels, which could contribute to the production of passive sounds when burrowing or foraging, exhibit high fidelity to their burrows and specific sand banks and, therefore, could affect local and regional scale spatial variability in the soundscape (Tien et al., 2017). This could potentially explain why a higher variability in the phonic richness associated with the D2c habitat was observed, although this would need to be confirmed with visual surveys. Although the faunal assemblages have discrepancies in their composition, they, in fact, involve three very closely related cluster groups (Cooper & Barry, 2017) and are all characterised by relatively low numbers of taxa, as opposed to the other nine assemblages identified in UK waters. In addition, the substrate types (mixed, muddy, sandy), which are described to be generally associated with each faunal cluster group (Cooper & Barry, 2017), do not align with the habitat types classified by the EUNIS habitat map (coarse/hard and sandy). Our results indicate that a distinction can be made both at the coarser-resolution EUNIS classifications and, to a lesser extent, at the finer-resolution faunal cluster level. While no significant differences were found between the phonic richness associated with the faunal clusters, this may be explained by (i) limited replication for cluster D2a, (ii) high variability among samples collected in D2c, and (iii) the fact that phonic richness primarily reflects vocalising fishes and larger mobile invertebrates. As such, it may only act as a reliable proxy for benthic infaunal clusters when the presence and activity of these soniferous taxa accurately track variability in the underlying in- and epifaunal communities. Ground-truth sediment samples, rather than modelled results, may also help unravel further differences between the acoustic biodiversity associated with benthic habitats.

### Acoustic indices

Correlations between specific acoustic indices, the benthic richness, habitat type, and phonic richness were found, which is in contrast with results from some previous studies (Minello et al., 2021; Mooney et al., 2020; Pieretti & Danovaro, 2020). Higher benthic richness was linked to a greater acoustic complexity index, more even frequency bands, and quieter recordings. Harder substrates and a higher phonic richness were associated with more even signals overall, although this was more random across time bands in the recordings. They also corresponded to louder recordings but displayed less variation across frequency bands compared to sandy substrates.

Fish sounds typically cover the low-frequency bands, while invertebrate snapping and scraping sounds typically cover a broadband spectrum. The sounds recorded in our samples had both low and higher peak frequencies, which explains a correlation across all frequency bands. Although not enough replicates were present to test for significant differences, the indices suggest that the soundscapes associated with the D2a and D2b faunal assemblages, which also have a relatively high benthic richness, are driven by the presence of both low-frequency fish sounds and higher invertebrate sounds. Importantly, these sounds are not necessarily produced by the in- or epifaunal assemblages themselves. Rather, they are more likely to originate from soniferous fishes and mobile invertebrates that may incidentally inhabit or utilise the same areas, as well as from passive sounds generated by the behaviour of soniferous and non-soniferous species.

A higher variability in the ACI was found for the D2c faunal cluster, which could be indicative of these habitats being more dynamic, with distinct sandbanks having been found associated with distinct fish and epifaunal assemblages (Kaiser et al., 2004). While our results indicate that amplitude is correlated with the richness and habitat type metrics, these values will be influenced by both the amount of sound produced and the distance of the sound from the recorder. Since information on the distance of the sound from the recorder is missing, it is not possible to know if the amplitude is a reliable indicator of richness. Although our results suggest that benthic richness could be monitored with computationally efficient acoustic indices such as the ACI, AEI, and M (and indices such as BI, H, TE, SE, and M could be used to monitor changes in habitat type and phonic richness), a larger dataset needs to be analysed before any conclusions can be drawn, especially considering previous studies have shown that these metrics do not correctly represent biodiversity trends (Bolgan et al., 2018; Mooney et al., 2020; Raick et al., 2023a, 2025; Sugai et al., 2026).

### Study design

As part of this pilot study, we selected a single time point from a large dataset which was originally collected for marine mammal monitoring. Although the Marine Directorate provided data from 20 locations, data from only eight were used in this study to maximise the amount of data from contrasting habitat types while minimising the effect of seasonal, noise, and weather conditions. April was chosen because this is when key fish species are likely to spawn in Scottish waters (FishBase, 2024) and therefore produce sound. April was also expected to have lower ambient noise levels, as wind conditions could be calmer (De Clippele & Risch, 2021). To further optimise data quality for this study, sound pressure levels were calculated to select a day with the least noise. Although this study found spatial differences in the acoustic data, to fully understand the usefulness of PAM data for longer-term monitoring programmes, further research is needed to investigate the implications of seasonal and weather (i.e. ambient noise) conditions.

We also wanted to assess whether noise pollution may negatively affect the usability of easy-to-calculate acoustic indices. Our analysis suggests that statistically speaking, correlations can be found, but that noise pollution can affect the observed patterns and that, therefore, these indices need to be used with care. Self-noise (i.e. mooring noise), but also noise pollution as a consequence of pile driving and shipping, affected the length of the recordings that were usable for the analysis of types of sounds and acoustic indices. Arbroath was the most heavily affected site, followed by Latheron and Fraserburgh. Pile-driving signals were detected at both the Cruden Bay and Arbroath sites, with the signal being particularly prominent at Arbroath. Although records of construction activities in the region were available, the exact source of these sounds could not be confidently identified. Potential contributors include construction activities associated with harbour infrastructure works in the region (see SM7 and SM8 for more information). Only a small percentage (~ 10%) of the recording length of the samples from the other locations were affected. Regardless of their origin, noise pollution could mask the presence of sounds and could, therefore, negatively affect the number of sounds detected in the file. Furthermore, through masking biological sounds and through their presence and alteration, they can affect the performance of the acoustic indices. When comparing the acoustic indices calculated on the raw and cleaned files, no significant differences were found. However, this does not rule out that the acoustic index values can be drastically different for a file which has anthropogenic noise removed. For example, in Fig. 5, it can be observed that the ACI pattern is different and that the value for the D2a faunal cluster is much higher in the cleaned vs the uncleaned data. No different pattern was observed for the BI values related to hard and sandy habitat types, but again, the values for the cleaned data are much higher. While accounting for differences in sample length as a result of removing noise events may improve comparability, we found that values still differed, and location- or habitat-specific differences exist. We therefore conclude that it is not advisable to draw conclusions when comparing metrics from cleaned datasets with those that are uncleaned. Furthermore, since noise pollution can affect sample length and mask sounds, PAM to assess changes in biodiversity in heavily noise-polluted samples or sites may not be feasible. In addition to this, it is not advisable to compare the values of different indices between locations or timelines, as the influence of different levels of noise pollution cannot be detangled in their response. The best practice would be to minimise mooring noise during installation, quality-check the data for high levels of mooring or pile-driving noise, and remove them where possible while accounting for differences in the sample length in the data analysis.

Although this study did not aim to describe temporal patterns, here the phonic richness was on average twice as high at sunrise and 1.7 times higher at sunset times, compared to midday, suggesting a higher soniferous activity at these times. Circadian rhythms have been observed to drive changes in marine soundscapes in temperate and tropical ecosystems, with increased activity of fish sounds being present at dawn and dusk (Lamont et al., 2022; Siddagangaiah et al., 2021). However, because this study did not include temporal replications at each of the sites, these results need to be interpreted with care. Further studies should include a higher temporal replication and could reveal when, where and why these temporal patterns occur in offshore waters, which could, for example, also reveal information on the presence and duration of spawning events (Caiger et al., 2020; Fudge & Rose, 2009). Understanding these dynamics would enhance the ecological interpretation of soundscapes and strengthen their application for biodiversity monitoring and management.

### Further research needed to establish PAM as a benthic biodiversity monitoring tool

To fully establish the use of PAM as a tool to monitor benthic biodiversity, we recommend following three key steps. First, to overcome the time-consuming task of annotating and removing noise pollution from samples to calculate soundscape metrics, signal processing detectors or machine learning models could be developed to automatically detect and classify sound types in large acoustic datasets (Haver et al., 2023). Characterising sounds, as done in this study (SM5), is essential to allow for future identification of specific types of sounds and the development of detectors and models to increase the speed at which sounds are identified from large datasets. Second, this study only characterised the soundscapes associated with three faunal classes and included only a very small subset of the existing PAM data. The next step would be to characterise the soundscapes associated with all twelve clusters as identified by Cooper and Barry (2017), ideally while collecting additional visual or physical survey data to categorise the habitats and local diversity. Third, to distinguish natural from human- and climate change-driven changes, before-and-after impact (BACI) studies could be conducted, for example, assessing changes in the soundscape before, during and after the installation of an offshore windfarm. Timeseries analysis could then be used to understand how seasonal temperature versus climate change-driven temperature changes affect benthic soundscapes.

Our literature search indicated that at least 32 species, which are likely or potentially present at our sites, can make either passive or active sounds. The large majority of those potentially present, i.e. 234 species, are invertebrates, which are notoriously understudied for sonifery (Looby et al., 2023). As part of this study we published a database on Zenodo (De Clippele et al., 2025a) that provides the type of sound (as reported in the primary papers), min, max, and peak frequency, along with the number of pulses per second and length of the signature for the soniferous species found in the East Coast of Scotland. A total of 34 papers were reviewed. Since public databases were searched only for the presence of soniferous species within a 10-km radius of the moorings, future work could include a nationwide search to develop a comprehensive database and identification keys, which will help better understand the proportions of fish and invertebrates that make sound in the UK. While phonic richness does not provide an exact count of individuals or species, it serves as an indicator of the presence or absence of certain sound-producing animals, offering an indirect measure of overall community diversity (Desiderà et al., 2019; Lamont et al., 2022). It is important to note that each unique sound type does not always correspond to a single species, as some fish can produce multiple types of sounds for activities such as mating, aggression, or hunting (Banse et al., 2024a, 2024b; Colleye & Parmentier, 2012). However, the richness, abundance, and diversity of fish sounds, when measured using active sounds and traditional ecological indices (e.g. Shannon and Simpson), reflect not only fish taxonomic diversity but also the diversity of habitat use and behavioural activities within a site. Even when individual sounds cannot be confidently assigned to species, a habitat supporting many sound types likely hosts multiple behaviours (e.g. mating, foraging, nursery use), providing valuable insight into the ecological value and functional richness of that habitat, thus providing relevant information for managers (Desiderà et al., 2019).

PAM has the potential to support both species-specific and ecosystem-level biodiversity assessments. The development of machine learning classifiers for species-specific sounds would enable PAM to track the presence and activity of individual species and thus complement traditional ecosystem-biodiversity-based monitoring approaches (Caiger et al., 2020; Fudge & Rose, 2009; Pieretti & Danovaro, 2020). Furthermore, PAM can be used to reveal changes in environmental conditions as it has been linked to changes in wind, waves, and current speed conditions as well as anthropogenic activity measures such as noise pollution (De Clippele & Risch, 2021; Duarte et al., 2021; Merchant et al., 2016). Together, these applications highlight PAM’s dual role as a powerful tool for both ecological and environmental monitoring, strengthening its relevance for long-term ecosystem assessments.

To understand how offshore benthic habitats are changing, a variety of benthic tools and technologies are being used, each of which has pros and cons (Scottish Government Report, 2024). In addition to established tools, this pilot study demonstrates that PAM has the potential to be used as a generic benthic biodiversity indicator in sedimentary habitats, typically found around offshore wind farms. However, to ensure PAM can be used to monitor specific species, more data across seasonal, weather, and noise pollution conditions would need to be analysed and species-specific sounds would need to be identified and characterised. While the sounds made by some soniferous species have been characterised based on aquarium recordings, there is a lack of in situ “wild” recordings of animals in their natural environment, with natural background sounds. To increase our knowledge of which species makes which sound, audio-video arrays can be deployed, which allow one to associate sounds with specific species and behaviours (Mouy et al., 2023). This approach would also help determine which sounds are either passive or active. An in-depth understanding of the different species-specific types of sounds has the potential to reveal changes in the functional use and behaviour of species associated with a specific location, such as delineating spawning areas based on the presence of fish choruses (Stratoudakis et al., 2024) and monitoring migration patterns (Caiger et al., 2020).

Through passive acoustic recorders’ ability to collect data over long periods of time, at a high temporal resolution, without negatively impacting the environment, this cost-effective tool could be used to fill in gaps in our understanding regarding how benthic diversity and habitat use change over diel, seasonal, and inter-annual patterns across local and regional spatial scales. Future research could involve the refinement of fish detectors (Mouy et al., 2024) or species-specific machine learning classifiers (Caiger et al., 2020) to automatically analyse large datasets. Since certain sounds are linked to behaviour such as spawning or aggression, they could reveal more information on habitat use by fish and crustaceans (Caiger et al., 2020). However, it may be more challenging to use PAM data to reveal information on other metrics such as age and growth rate which are needed for fish stock assessments. In addition, the long-term and high-resolution nature of PAM generates very large datasets, creating challenges for data storage, management, and accessibility; however, advances in data infrastructures (e.g. use of compressed.sud instead of.wav files), open-access repositories (Looby et al., 2023), and databases (Darras et al., 2025) could help overcome these barriers and enhance the broader use of acoustic data.

Long-term marine monitoring is needed to inform management decisions to reduce the impacts of human activities and pressures (Borja & Elliott, 2021). This is especially relevant to Scotland, investing in a total of 27.6 GW for potential new offshore wind developments. The introduction of hard substrates, changing hydrodynamics, local primary productivity, and activities such as dredging and pile driving have the potential to impact benthic and benthopelagic communities at a local and regional scale (Wilhelmsson et al., 2006; Clark et al., 2014; Scott et al., 2018; Degraer et al., 2020; De Borger et al., 2021; Buyse et al., 2023a, 2023b; Knorrn et al., 2024).

## Conclusions

This study shows that existing PAM data collected for marine mammal monitoring can be used to infer differences in benthic biodiversity and substrate type. We discuss this study’s limitations and the range of research that can be undertaken to establish PAM as a monitoring tool for offshore benthic habitats. This could help understand how benthic biodiversity has changed over the last 10 years and serve as a baseline for future change resulting from offshore wind developments and climate change. Although we conclude that the acoustic indices cannot be used reliably, PAM can be used to detect differences in habitat types; it also has the potential to serve as early-warning indicators for temporal monitoring, provide behavioural information (e.g. spawning, feeding), or allow us to detect cryptic or undersampled species. In addition, the use of PAM as a cost-effective tool would also reduce our reliance on ships, which would contribute to the UK net zero goal to reduce carbon emissions (Liu et al., 2024). In light of recent discussions regarding Marine Net Gain (Hooper et al., 2021), PAM could also potentially be used as a tool to provide the metrics needed to measure biodiversity and functioning in areas associated with recovery or restoration activities.

## Supplementary Information

Below is the link to the electronic supplementary material.

ESM1 (DOCX 1.25 MB)

ESM2 (XLSX 129 KB)

## Data Availability

An overview of the list of soniferous species, along with their acoustic characteristics and the data used in the statistical analysis, along with the script, is available on Zenodo (De Clippele et al., 2025a). The raw PAM data can be requested from the Marine Directorate.
