# Supplementary material for: Passive acoustic monitoring (PAM) to assess benthic communities associated with offshore wind farms: insights and future directions
Source: Environ Monit Assess. 2026 Jun 29;198(7):783. doi: 10.1007/s10661-026-15614-5 (PMC13314827; doi:10.1007/s10661-026-15614-5)
Supplement: Supplementary file 1 — (DOCX 1.25 MB) [file 10661_2026_15614_MOESM1_ESM.docx]

# **Supplementary materials**

### **SM 1 Sound Pressure Levels**

Sound Pressure Levels were calculated using PAMGuide in MATLAB (Merchant et al. 2015). Third-Octave Level (TOL) sound pressure levels were calculated at 60-second resolution from 1-2000Hz, in order to characterise both benthic biodiversity and noise pollution from shipping noise (Merchant et al. 2016). TOL data was calculated based on the raw sound files. While some shipping activity is present at the St Abbs location (SPL peaks at 130 dB and 120 dB), the 10^th^ of April is mostly absent of ship noise. H100 indicates the third-octave band centred at 100 Hz (dB).


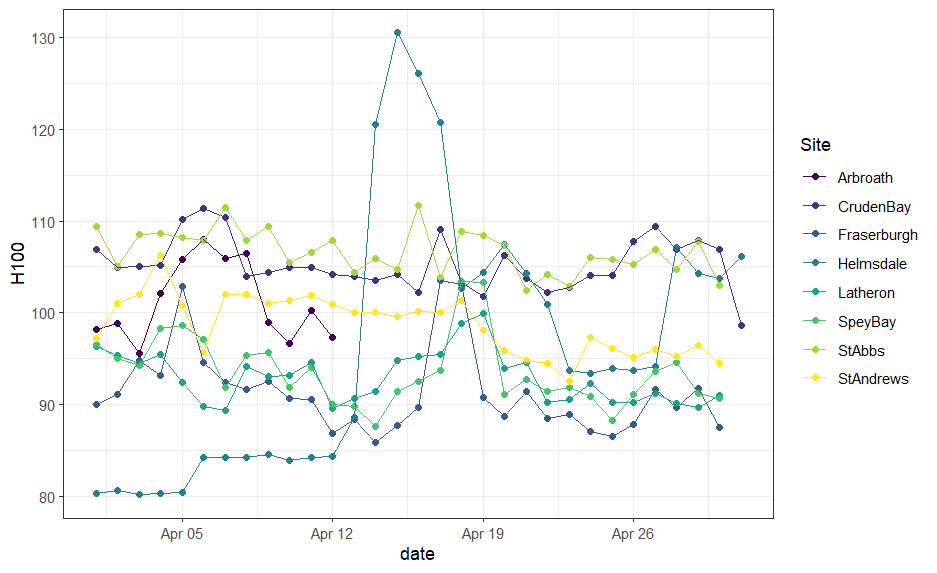


Daily average SPL data for the 100Hz third-octave level across all 8 moorings. April 10^th^ is the best date for covering all sites, without high levels of shipping noise.


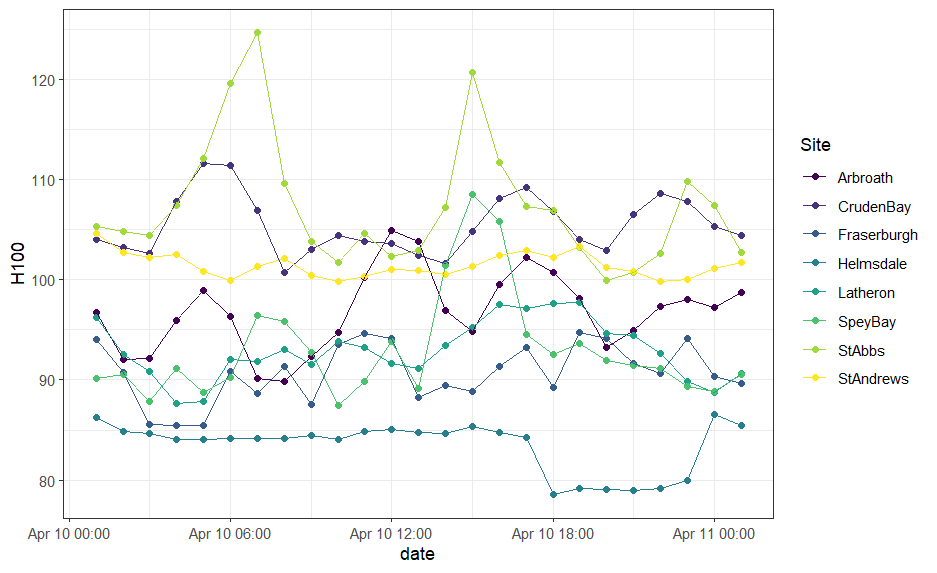


Hourly average SPL for 100Hz third-octave level on April 10^th^ 2021.

### **SM 2 Biological and environmental data sets**

Overview of the contextual and environmental data layers, which were downloaded from open-source websites. NA: Not applicable as this is a shapefile. For full description of the variables, please explore the source.

| Layer | Resolution | Source |
| --- | --- | --- |
| Depth (m) | 15 arc second (~460x265 m) | <https://gebco.net/data_and_products/gridded_bathymetry_data/> |
| Habitat map (EUNIS classification) | NA | <https://hub.jncc.gov.uk/assets/202874e5-0446-4ba7-8323-24462077561e#C20220322-UKSeaMap2018-WGS84.zip> |
| Temperature (°C), Current velocity (m^-s^), Light (-), Dissolved molecular oxygen (mmol m^-3^), Phytoplankton (µmol m^-3^), Primary Productivity (g m^-3^ day^-1^), Chlorophyll (mg m^-3^) | 0.05° resolution (~5.55 x 3.18 km) | <https://bio-oracle.org/> version 2.2, present-day conditions (2000-2014), benthic layers |
| Faunal cluster class, benthic biodiversity | NA | <https://rconnect.cefas.co.uk/onebenthic_portal/> |
| NBN atlas | NA | <https://nbnatlas.org/> |
| Spawning and nursery ground layers | NA | <https://data.cefas.co.uk/view/153>  <https://www.gov.scot/publications/developing-essential-fish-habitat-maps-fish-shellfish-species-scotland-report/pages/3/> |

### **SM 3 Start time and length of the recordings**

The timings which are highlighted in bold indicate the files that had the largest amount of noise pollution and were therefore drastically reduced in file length.

| Site | Phase | Start time | Cleaned file length (mm:ss) |
| --- | --- | --- | --- |
| Arbroath | Midnight | 01:20:00 | **04:12** |
| Arbroath | Sunrise | 06:20:00 | **03:16** |
| Arbroath | Midday | 13:20:00 | **02:43** |
| Arbroath | Sunset | 19:50:00 | **04:02** |
| CrudenBay | Midnight | 01:25:00 | 09:50 |
| CrudenBay | Sunrise | 06:25:00 | 09:56 |
| CrudenBay | Midday | 12:55:00 | 09:46 |
| CrudenBay | Sunset | 19:55:00 | 09:40 |
| Fraserburgh | Midnight | 01:05:00 | **05:31** |
| Fraserburgh | Sunrise | 06:35:00 | 09:17 |
| Fraserburgh | Midday | 13:05:00 | **06:02** |
| Fraserburgh | Sunset | 20:05:00 | 09:40 |
| Helmsdale | Midnight | 01:25:00 | 09:59 |
| Helmsdale | Sunrise | 06:25:00 | 09:59 |
| Helmsdale | Midday | 13:25:00 | 09:59 |
| Helmsdale | Sunset | 19:55:00 | 09:59 |
| Latheron | Midnight | 01:15:00 | **06:27** |
| Latheron | Sunrise | 06:15:00 | 09:38 |
| Latheron | Midday | 13:15:00 | **04:29** |
| Latheron | Sunset | 19:45:00 | 06:50 |
| SpeyBay | Midnight | 01:15:00 | 09:58 |
| SpeyBay | Sunrise | 06:15:00 | 09:59 |
| SpeyBay | Midday | 13:15:00 | 09:40 |
| SpeyBay | Sunset | 19:45:00 | 10:00 |
| StAbbs | Midnight | 01:00:00 | 09:27 |
| StAbbs | Sunrise | 06:30:00 | 10:00 |
| StAbbs | Midday | 13:00:00 | 09:18 |
| StAbbs | Sunset | 20:00:00 | 09:09 |
| StAndrews | Midnight | 01:00:00 | 09:58 |
| StAndrews | Sunrise | 06:30:00 | 09:53 |
| StAndrews | Midday | 13:00:00 | 10:00 |
| StAndrews | Sunset | 20:00:00 | 09:52 |

### Information regarding the deployment number, location, hydrophone, sensitivity, gain, duty cycle and location. LS1x Loggerhead instruments were used.

| No. | Location | Loggerheadhydrophone | Sensitivity | Gain | Duty Cycle | Lat DD | Lon DD | Depth (m) |
| --- | --- | --- | --- | --- | --- | --- | --- | --- |
| 554 | Latheron 5 | LS1x-23 | -169.85 | 12.4 | 10 on / 20 off | 58.27 | -3.32 | -26 |
| 523 | Helmsdale 15 | LS1X-27 | -170.7 | 12.4 | 10 on / 20 off | 57.98 | -3.54 | -48 |
| 549 | Spey Bay 10 | LS1x-22 | -169.44 | 12.4 | 10 on / 20 off | 57.74 | -3.05 | -23 |
| 530 | Fraserburgh 5 | LS1-1 | -175.85 | 12.4 | 10 on / 20 off | 57.71 | -2.13 | -38 |
| 533 | Cruden Bay 5 | LS1-8 | -175.44 | 12.4 | 10 on / 20 off | 57.38 | -1.83 | -21 |
| 546 | Arbroath 10 | LS1x-28 | -170.43 | 12.4 | 10 on / 20 off | 56.5 | -2.38 | -47 |
| 543 | St Andrews 10 | LS1x-21 | -170.42 | 12.4 | 10 on / 20 off | 56.26 | -2.5 | -41 |
| 536 | St Abbs 5 | LS1x-18 | -169.92 | 12.4 | 10 on / 20 off | 55.93 | -2.18 | -34 |

### **SM 4 Biological variables associated with each site**

| **Location** | **Predicted Habitat type** | **Depth (m)** | Temp (°C) | Current velocity (m-^s^ ) | Dissolved organic carbon (mmol m^-3^) | Primary Productivity (g m^-3^ day^-1^) | **Taxa Richness (average ± stdev)** | **Faunal Class** | **Area Class (km^2^)** | **No. Taxonomic Classes** |
| --- | --- | --- | --- | --- | --- | --- | --- | --- | --- | --- |
| Spey Bay | Coarse | 23 | 9.638 | 0.073 | 285.318 | 0.0066 | 8±5 | D2c | 136.66 | 5 |
| Latheron | Coarse | 26 | 9.576 | 0.038 | 278.575 | 0.0030 | 24±8 | D2a | 30.69 | 3 |
| Fraserburgh | Coarse | 38 | 8.593 | 0.112 | 262.774 | 0.0000 | 27±8 | D2b | 147.57 | 3 |
| St Andrews | Coarse | 41 | 9.037 | 0.040 | 274.008 | 0.0011 | 27±8 | D2b | 152.59 | 6 |
| St Abbs | Hard | 34 | 8.535 | 0.044 | 264.779 | 0.005 | 8±5 | D2c | 17.86 | 5 |
| Cruden Bay | Sandy | 21 | 8.811 | 0.072 | 270.153 | 0.0006 | 8±5 | D2c | 152.72 | 5 |
| Arbroath | Sandy | 47 | 8.894 | 0.036 | 271.126 | 0.0008 | 24±8 | D2b | 65.13 | 5 |
| Helmsdale | Sandy | 48 | 9.539 | 0.030 | 271.587 | 0.0012 | 24±8 | D2b | 295.15 | 5 |

### **SM 5 Acoustic characteristics of the types of sounds**

Presented are the Centre Frequency (Freq), Peak Frequency, Frequency Contour Percentile 25% (Freq 25%) Period duration (Delta Time), Period Pulse period and the number of pulses. The average (av) and standard deviation (stdev) values are provided. Here, a good signal-to-noise ratio (SNR) refers to signals that were sufficiently distinguishable from background noise to allow reliable measurement of the acoustic metrics listed in this table.

| Type of Sound | No Samples with good SNR | Centre Freq (Hz) | | Freq 25% (Hz) | | Freq 95% (Hz) | | Peak Freq (Hz) | | Sound Duration (s) | | Pulse period (s) | | Number of pulses | |
| --- | --- | --- | --- | --- | --- | --- | --- | --- | --- | --- | --- | --- | --- | --- | --- |
|  |  | av | stdev | av | stdev | av | stdev | av | stdev | av | stdev | av | stdev | av | stdev |
| Chirp | 4 | 1500 | 153 | 1406 | 108 | 1688 | 153 | 1547 | 94 | 0.020 | 0.005 | 0.005 | 0.001 | 3.0 | 2.0 |
| Crackle | 9 | 3625 | 162 | 3229 | 83 | 4167 | 83 | 3917 | 113 | 1.200 | 0.858 | 0.136 | 0.062 | 9.0 | 7.9 |
| Drum | 53 | 1065 | 36 | 1010 | 61 | 1238 | 51 | 1068 | 47 | 0.018 | 0.013 | 44.070 | 220.311 | 1.8 | 1.2 |
| Grunt | 2 | 23 | 0 | 18 | 8 | 94 | 66 | 18 | 8 | 0.260 | 0.108 | 0.042 | 0.000 | 6.5 | 2.1 |
| Hard Grunt | 0 | NA | NA | NA | NA | NA | NA | NA | NA | NA | NA | NA | NA | NA | NA |
| Plop | 5 | 61 | 84 | 14 | 31 | 281 | 29 | 66 | 94 | 0.039 | 0.018 | NA | NA | 1.0 | 0.0 |
| Scrape | 8 | 788 | 16 | 738 | 24 | 974 | 61 | 782 | 29 | 0.390 | 0.463 | 0.212 | 0.132 | 3.3 | 3.3 |
| Snap | 3 | 3188 | 5521 | 1813 | 3139 | 6125 | 9016 | 125 | 217 | 0.003 | 0.002 | NA | NA | 1.0 | 0.0 |
| Sneeze | 1 | 586 | NA | 328 | NA | 3703 | NA | 281 | NA | 0.204 | NA | NA | NA | 1.0 | NA |
| Squeak | 4 | 1354 | 67 | 1242 | 47 | 1764 | 82 | 1330 | 177 | 0.015 | 0.013 | NA | NA | 1.0 | 0.0 |
| Tap | 11 | 158 | 111 | 100 | 109 | 303 | 30 | 124 | 129 | 0.029 | 0.018 | 0.041 | 0.021 | 1.2 | 0.4 |
| Thump | 6 | 0 | 0 | 0 | 0 | 188 | 0 | 0 | 0 | 0.289 | 0.063 | 0.182 | 0.021 | 1.3 | 0.5 |
| Tick | 14 | 10031 | 263 | 9723 | NA | 10795 | 160 | 10085 | 472 | 0.004 | 0.002 | 0.003 | NA | 1.1 | 0.3 |
| Whew | 1 | 1125 | NA | 961 | NA | 1383 | NA | 961 | NA | 0.176 | NA | NA | NA | 1.0 | NA |
| Whoop | 4 | 398 | 27 | 387 | 23 | 516 | 38 | 410 | 45 | 0.043 | 0.016 | 0.024 | 0.010 | 2.0 | 0.0 |
| Whoosh | 1 | 844 | NA | 797 | NA | 984 | NA | 844 | NA | 0.053 | NA | 0.011 | NA | 4.0 | NA |

### **SM 6 Spectrogram (left) and waveforms (right) of the types of sounds**

**Chirp**


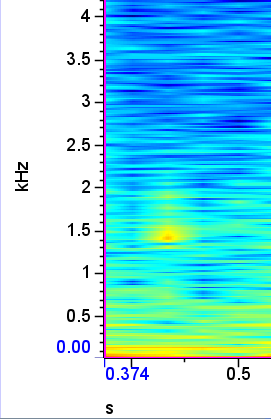

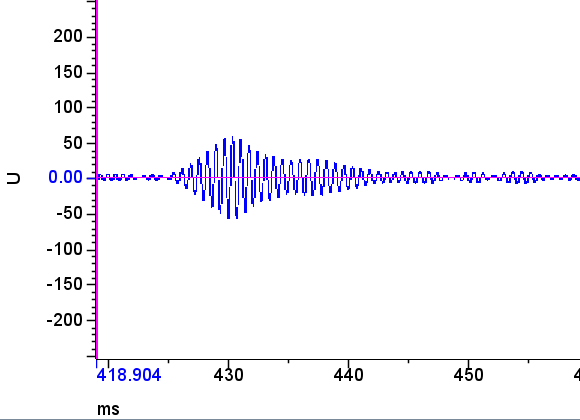


**Crackle**


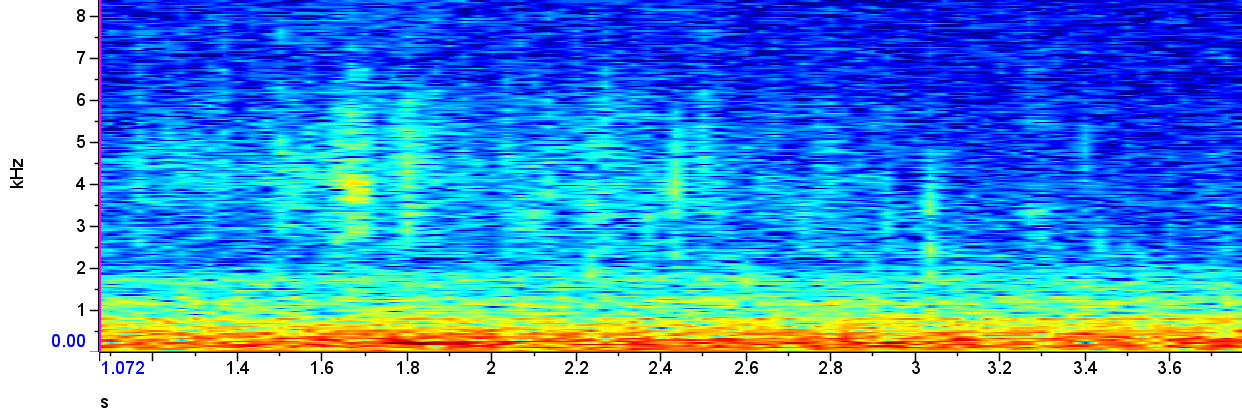

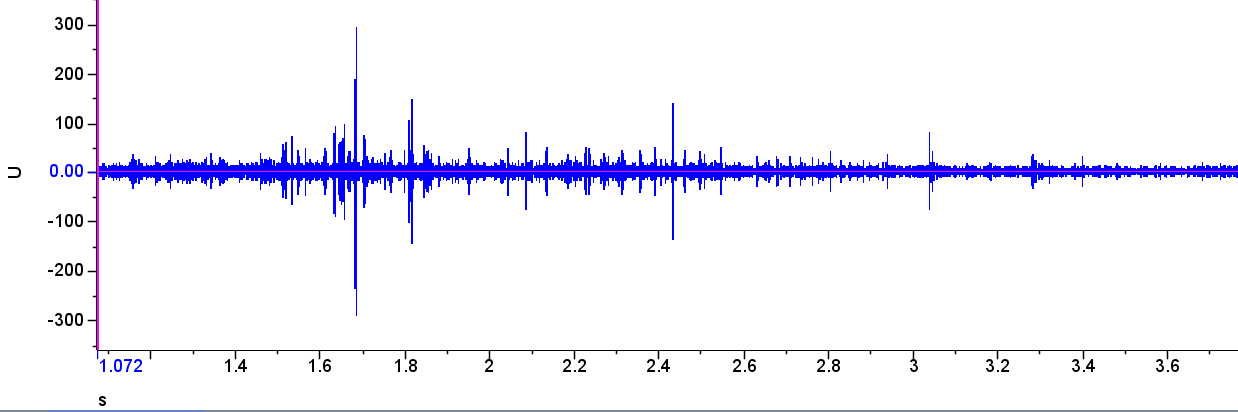


**Drum**


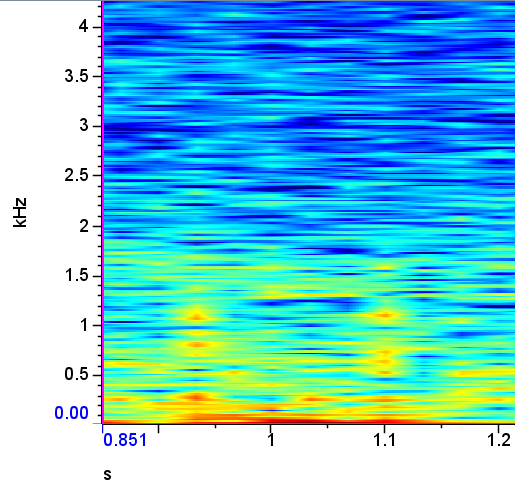

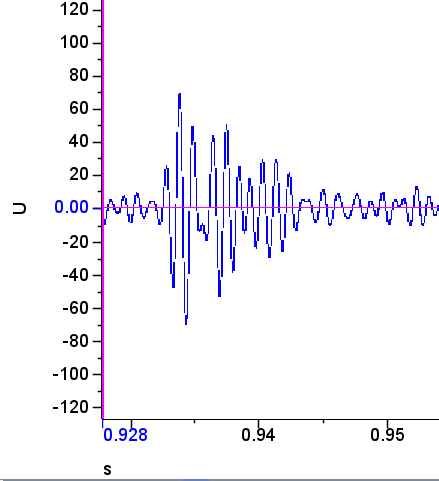


**Grunt**


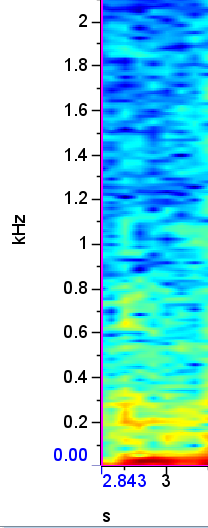

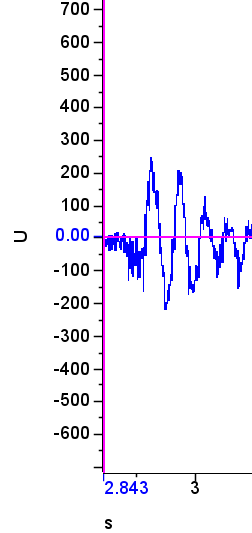


**Plop**


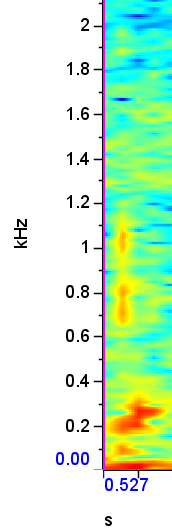

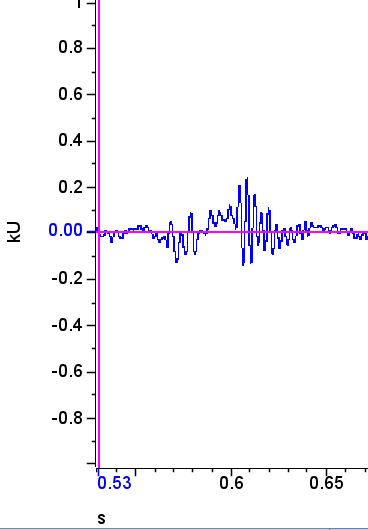


**Scrape**


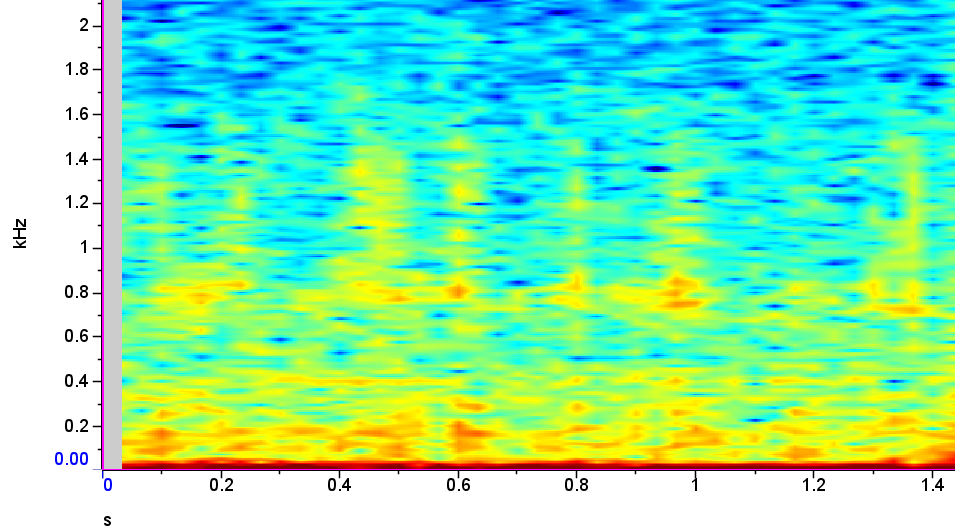

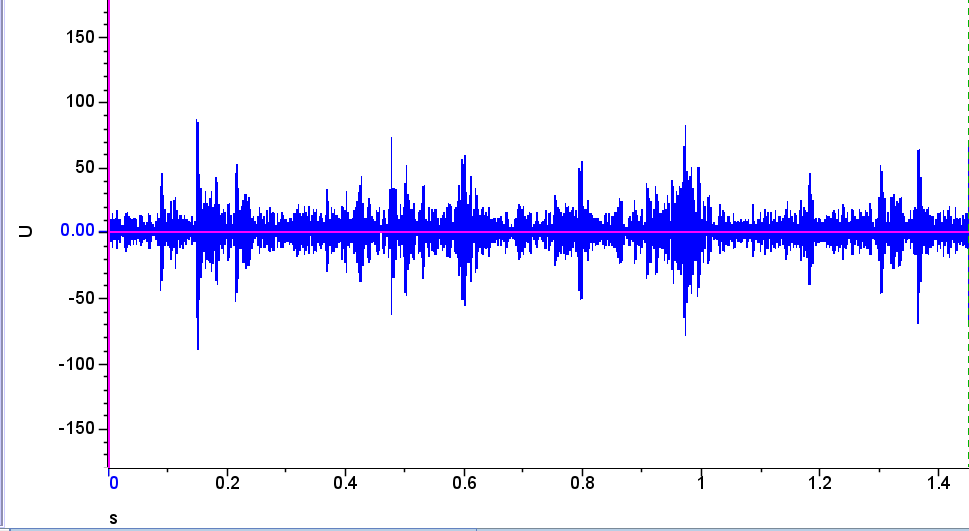


**Snap**


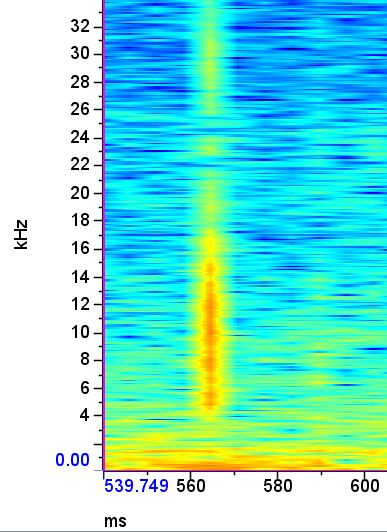

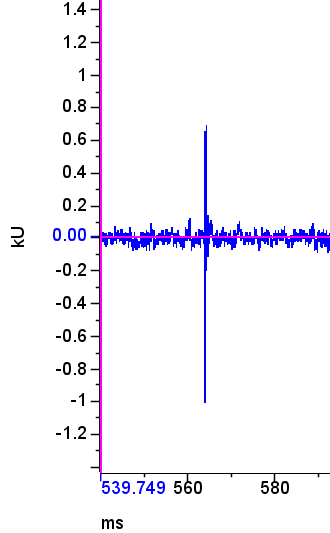


**Sneeze**


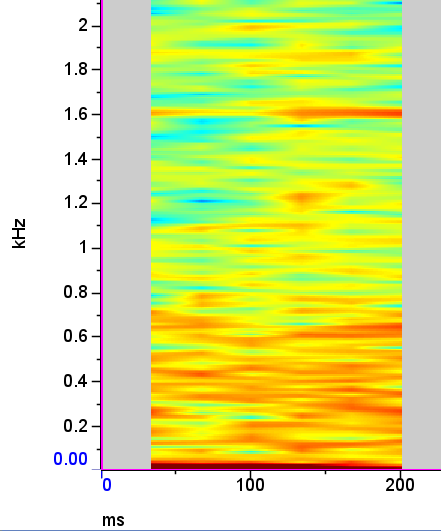

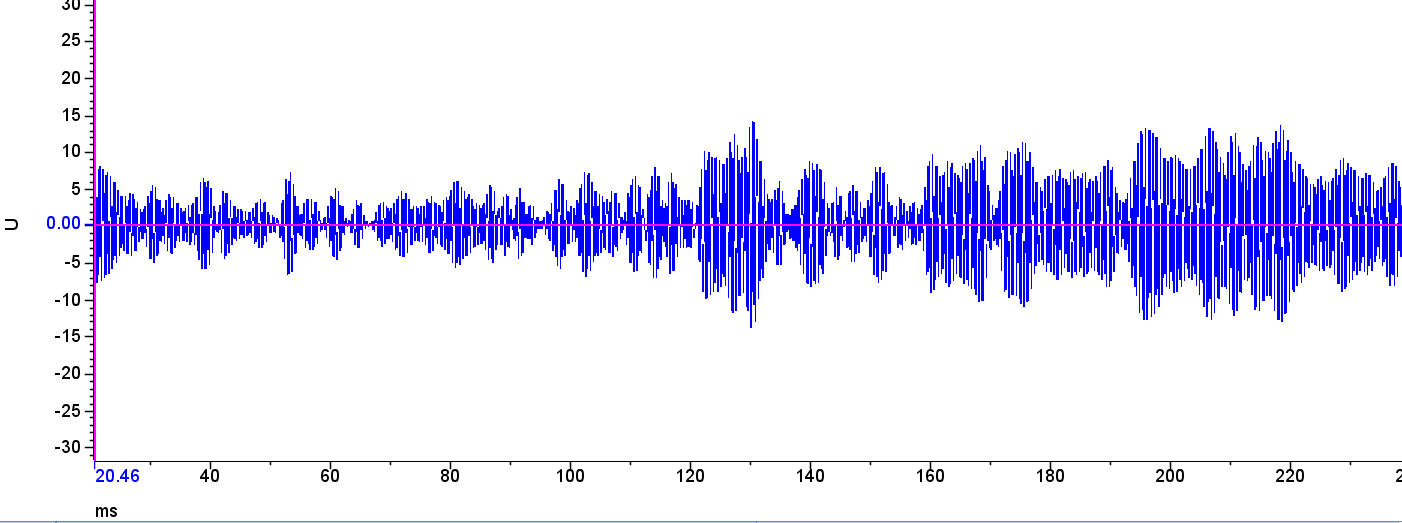


**Squeak**


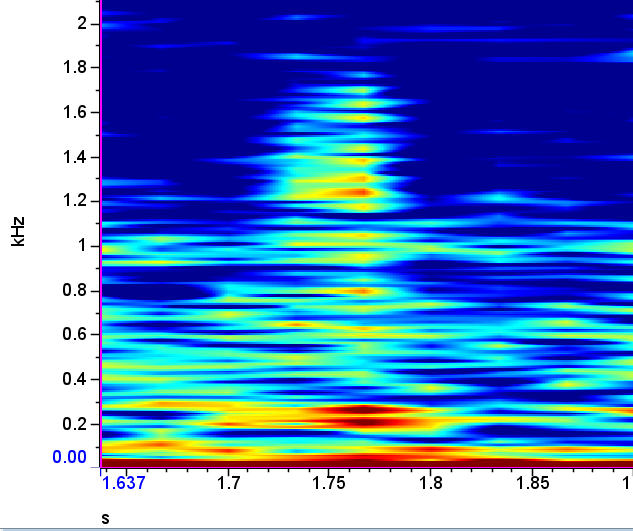

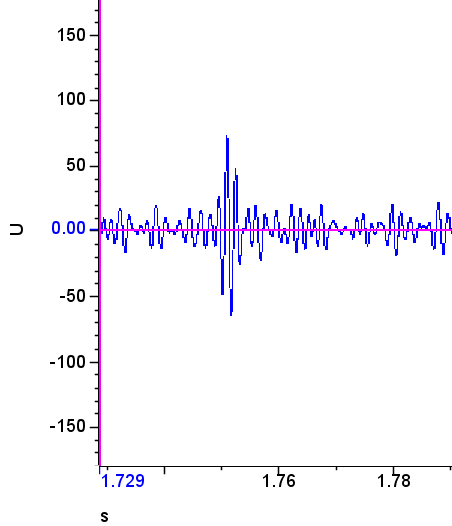


**Tap**


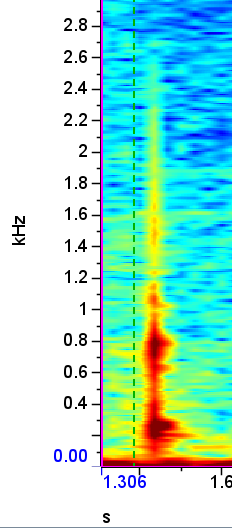

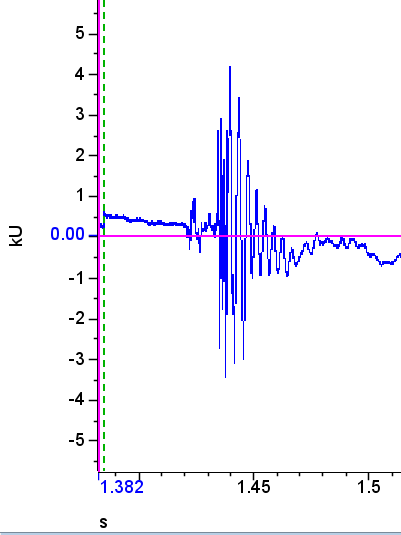


**Thump**


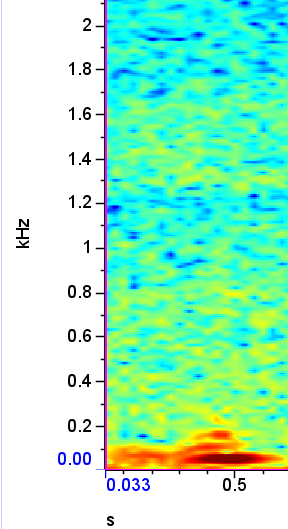

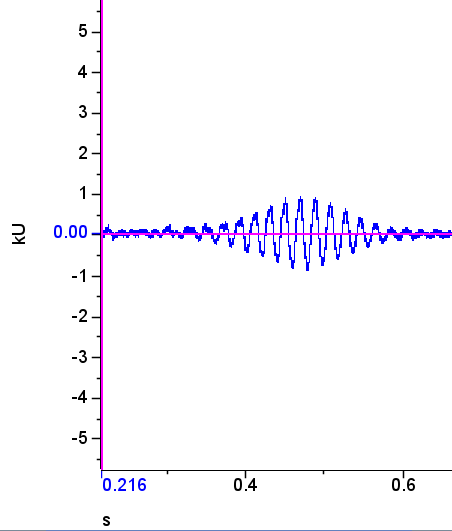


**Tick**


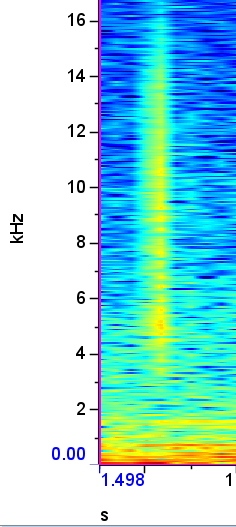

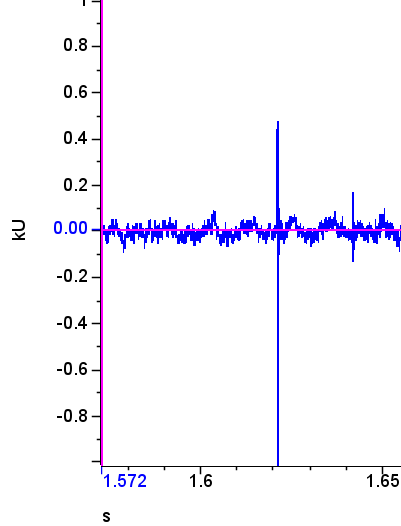


**Whew**


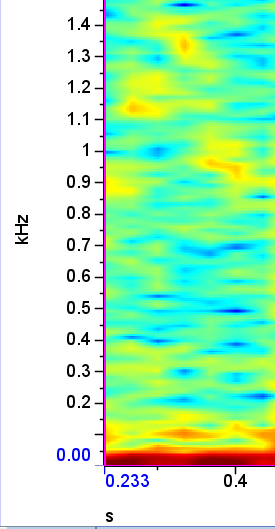

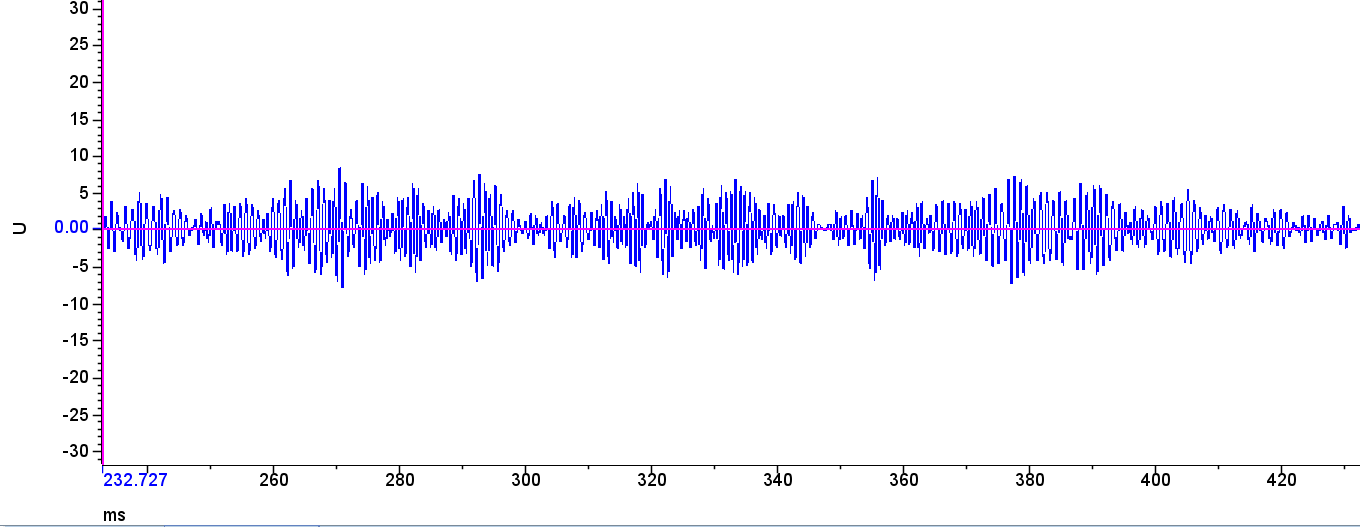


**Whoop**


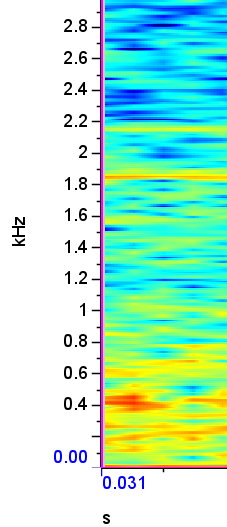

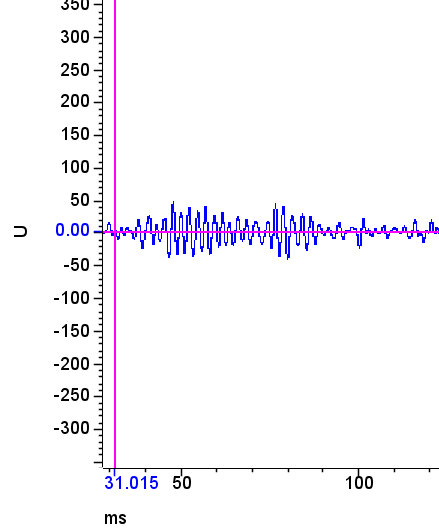


**Woosh**


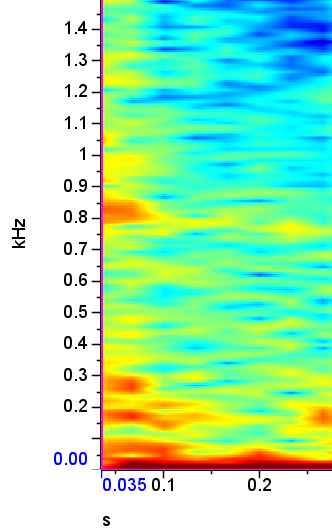

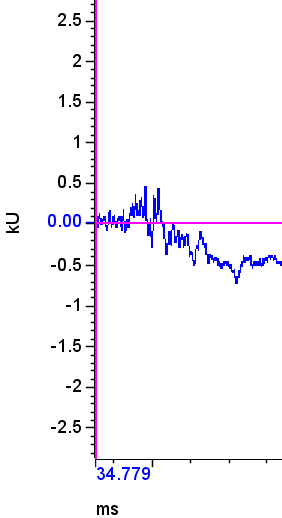


**SM7: Origin of pile driving sounds**

Pile-driving signals were observed at Cruden Bay and Arbroath, with the signal being clearer at Arbroath, suggesting a source closer to that location. Below is a table showing the year of operation for nearby wind farm sites. Neart na Gaoithe, Kincardine, and Seagreen and Morray East were closest to Cruden Bay and Arbroath. However, Neart na Gaoithe, Kincardine, and Seagreen projects did not undertake pile-driving activities during the relevant period, leaving the Moray East development as the most likely offshore wind farm source. However, Moray East is located at a considerable distance from both Cruden Bay and Arbroath, and pile-driving signals were not observed at other recording locations (e.g., Spey Bay and Fraserburgh) that are geographically closer to the Moray East site.

We also examined entries in the UK Marine Noise Registry (MNR) (see SM6). A record dated 10 April 2021 identified a pile-driving licence application associated with Dundee Port ([https://marine.gov.scot/sites/default/files/marine_licence_application_form_-_construction.pdf](https://eur03.safelinks.protection.outlook.com/?url=https%3A%2F%2Fmarine.gov.scot%2Fsites%2Fdefault%2Ffiles%2Fmarine_licence_application_form_-_construction.pdf&data=05%7C02%7CLaurence.DeClippele%40glasgow.ac.uk%7C6b3b09ea7fe74bab0ed508debcbdc866%7C6e725c29763a4f5081f22e254f0133c8%7C1%7C0%7C639155720929283084%7CUnknown%7CTWFpbGZsb3d8eyJFbXB0eU1hcGkiOnRydWUsIlYiOiIwLjAuMDAwMCIsIlAiOiJXaW4zMiIsIkFOIjoiTWFpbCIsIldUIjoyfQ%3D%3D%7C0%7C%7C%7C&sdata=GVEFdacHhFV8HivsVmPO0bRJuVNprWVU7CteN3SVLsY%3D&reserved=0) ). While this activity could contribute to the observed signals, the distance from Dundee to the recording sites precludes confident attribution.

An alternative explanation is that the sounds originated from harbour construction activities closer to the recording locations. For example, a marine licence application for pontoon installation at Montrose Harbour ([marine_licence_application_redacted_40.pdf](https://eur03.safelinks.protection.outlook.com/?url=https%3A%2F%2Fmarine.gov.scot%2Fsites%2Fdefault%2Ffiles%2Fmarine_licence_application_redacted_40.pdf&data=05%7C02%7CLaurence.DeClippele%40glasgow.ac.uk%7C6b3b09ea7fe74bab0ed508debcbdc866%7C6e725c29763a4f5081f22e254f0133c8%7C1%7C0%7C639155720929249909%7CUnknown%7CTWFpbGZsb3d8eyJFbXB0eU1hcGkiOnRydWUsIlYiOiIwLjAuMDAwMCIsIlAiOiJXaW4zMiIsIkFOIjoiTWFpbCIsIldUIjoyfQ%3D%3D%7C0%7C%7C%7C&sdata=ce%2Bma12JpYRbPKHckeRwQaiiE%2BGWFYCdq0mMq%2F%2BPDwI%3D&reserved=0) ), included pile-driving operations. However, no noise was registered on the MNR for the date we analysed the recordings. While harbour-based construction may therefore represent a plausible source of the recorded signals, given the uncertainties, we cannot definitively attribute the observed signals to a particular project or location.

**Table** providing an overview of the offshore windfarms, type of installation, foundation type, number of turbines and the year of operation

| **Site** | **Fixed or Floating** | **Foundation type** | **Number of turbines** |  |  | **Start -end year of construction** |
| --- | --- | --- | --- | --- | --- | --- |
| BOWL | Fixed | Jacket | 84 |  |  | 2016-2018 |
| Hywind | Floating | Lines and moorings | 5 |  |  | 2016-2017 |
| Kincardine | Floating | Lines and moorings | 5 |  |  | 2018-2021 |
| Greater Gabbard | Fixed | Monopile | 140 |  |  | 2008-2012 |
| Humber Gateway | Fixed | Monopile | 73 |  |  | 2012-2015 |
| Seagreen | Fixed | Monopile | 114 |  |  | 2021-2022 |
| Robin Rigg | Fixed | Monopile | 60 |  |  | 2008-2010 |
| Blyth | Fixed | Gravity-based | 5 |  |  | 2000 |
| Aberdeen | Fixed | Suction bucket jackets | 11 |  |  | 2018 |
| Moray East | Fixed | Jacket | 100 |  |  | 2019-2021 |
| Neart na Gaoithe | Fixed | Jacket | 54 |  |  | 2020 – 2024 |
